# Recombinant SpTransformer proteins are functionally diverse for binding and phagocytosis by three subtypes of sea urchin phagocytes

**DOI:** 10.3389/fimmu.2024.1372904

**Published:** 2024-04-29

**Authors:** Ryley S. Crow, Chloe G. Shaw, Leon Grayfer, L Courtney Smith

**Affiliations:** Department of Biological Sciences, George Washington University, Washington, DC, United States

**Keywords:** *Strongylocentrotus purpuratus*, polygonal phagocyte, discoidal phagocyte, small phagocyte, inert beads

## Abstract

**Introduction:**

The California purple sea urchin, *Strongylocentrotus purpuratus*, relies solely on an innate immune system to combat the many pathogens in the marine environment. One aspect of their molecular defenses is the *SpTransformer* (*SpTrf*) gene family that is upregulated in response to immune challenge. The gene sequences are highly variable both within and among animals and likely encode thousands of SpTrf isoforms within the sea urchin population. The native SpTrf proteins bind foreign targets and augment phagocytosis of a marine *Vibrio*. A recombinant (r)SpTrf-E1-Ec protein produced by *E. coli* also binds *Vibrio* but does not augment phagocytosis.

**Methods:**

To address the question of whether other rSpTrf isoforms function as opsonins and augment phagocytosis, six rSpTrf proteins were expressed in insect cells.

**Results:**

The rSpTrf proteins are larger than expected, are glycosylated, and one dimerized irreversibly. Each rSpTrf protein cross-linked to inert magnetic beads (rSpTrf::beads) results in different levels of surface binding and phagocytosis by phagocytes. Initial analysis shows that significantly more rSpTrf::beads associate with cells compared to control BSA::beads. Binding specificity was verified by pre-incubating the rSpTrf::beads with antibodies, which reduces the association with phagocytes. The different rSpTrf::beads show significant differences for cell surface binding and phagocytosis by phagocytes. Furthermore, there are differences among the three distinct types of phagocytes that show specific vs. constitutive binding and phagocytosis.

**Conclusion:**

These findings illustrate the complexity and effectiveness of the sea urchin innate immune system driven by the natSpTrf proteins and the phagocyte cell populations that act to neutralize a wide range of foreign pathogens.

## 1 Introduction

Innate immune systems function with a variety of pathogen recognition receptors (PRRs) that are located on the cell surface, the endomembrane system, in the cytoplasm, or secreted into the extracellular fluid. Well studied PRRs include Toll-like receptors (TLRs) (1–3), NOD-like receptors (NLRs) (4, 5), RIG-I-like receptors (RLRs) (6, 7), C-type lectin receptors (CLRs) (8), as well as resistance (R) proteins in plants (9, 10). PRR recognition of immunological insult is the core of all metazoan defenses and have key functions in invertebrate species, which lack immune receptor genes that are built by somatic recombination or copy choice that confer adaptive immunity in vertebrates (11–13). In response to PRR signaling, organisms secrete a wide variety of effector proteins that include expanded gene families encoding anti-microbial peptides and proteins (AMPs) (14–17). Invertebrates such as sea urchins have a sophisticated innate immune system with expanded gene families encoding TLRs, NLRs [(18, 19); reviewed in (20, 21)], small C-type lectins (19), and a variety of AMPs that function in a wide range of organisms (16, 22, 23). The purple sea urchin from the Pacific coast of North America, *Strongylocentrotus purpuratus*, also has the *SpTransformer* (*SpTrf*) gene family [reviewed in (24)] that encodes unique immune effector proteins. The *Trf* genes are found exclusively in euechinoids and have been reported in purple sea urchins in Australia, *Heliocidaris erythrogramma*, *HeTrf* (25) and in the Mediterranean, *Paracentrotus lividus*, *PlTrf* (26). The *Trf* gene sequences are similar among the sea urchin species, but fall into phylogenetically separate clades (25, 26).

**Figure 1:**
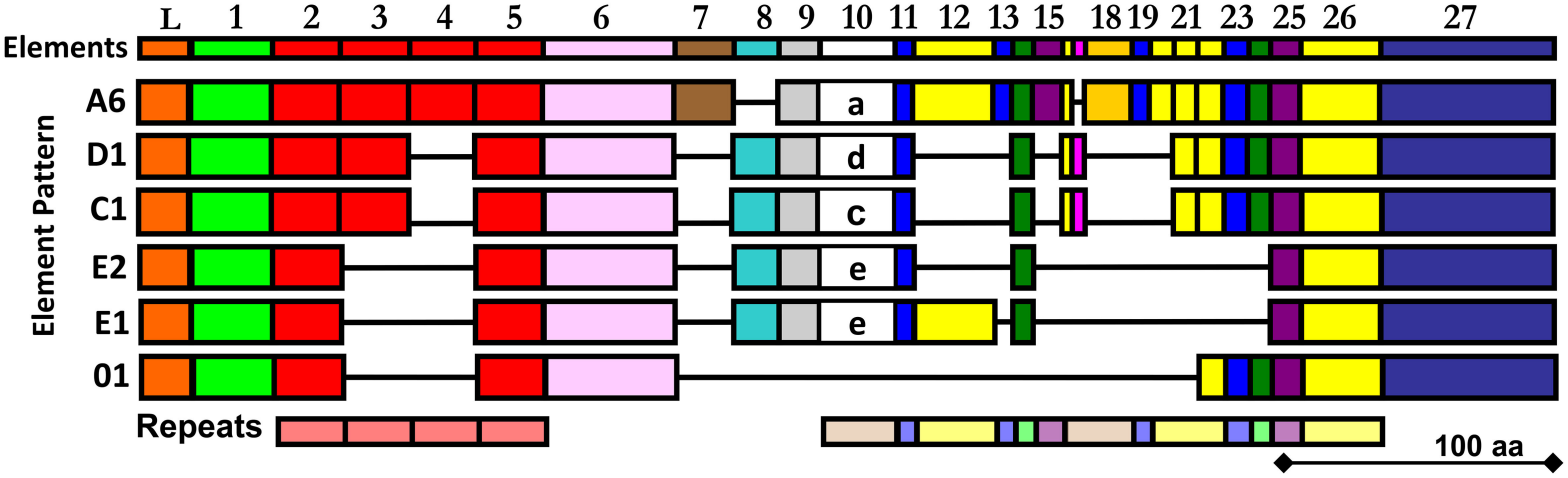
The alignment illustrates the mosaic element patterns in the rSpTrf proteins chosen for expression in insect cells. Elements are recognizable blocks of sequence that are slightly variable, and are defined by gaps in an alignment of all known SpTrf protein sequences deduced from cDNA and gene sequences (30, 31). Elements are depicted as colored rectangles. All possible elements are numbered at the top of the alignment. There are no SpTrf sequences that include all elements. Both tandem and interspersed repeats are shown below the alignment. This figure is modified from (32).

The *SpTrf* gene family was initially identified based on significantly elevated expression in response to challenge with marine bacteria (27) and to a variety of pathogen associated molecular patterns (PAMPs) (28–30). This expression pattern is consistent with immune response functions of the encoded proteins. Furthermore, full length sequences of the *SpTrf* cDNAs and genes show a significant level of sequence diversity, in part based on single nucleotide polymorphisms that translate to amino acid diversity. Optimized alignments require the insertion of large artificial gaps that define blocks of slightly variable sequences termed ‘elements’ (**Figure 1**) (30, 31, 33, 34). Elements of different sequence and length are present in the HeTrf and PlTrf proteins, although some of the repeats in the coding region are similar to those in SpTrf (25, 26). *In silico* analyses (DisMeta server (https://montelionelab.chem.rpi.edu/dismeta/)) predicts that the Trf proteins are all entirely disordered, which is supported by results from circular dichroism of one recombinant version (r)SpTrf-E1 expressed in *E. coli* (rSpTrf-E1-Ec) (35, 36) and by AlphaFold (DeepMind.com) for several Trf proteins.

The Trf proteins in *S. purpuratus* and *H. erythrogramma* are expressed by the phagocyte class of coelomocytes in response to immune challenge, with elevated expression in the polygonal, medium, and small phagocytes and much lower expression in discoidal phagocytes (25, 29, 37–39); [reviewed in (24)]. All Trf proteins have the same general structure of a leader followed by a glycine-rich region, a central multimerization region, a histidine-rich region, and a C terminal region (**Figure 1**; **Supplementary Figure S1**) and although all Trf proteins from all species multimerize irreversibly, their multimerization motifs are dissimilar (25, 26, 30, 35, 37, 40). As indicated by the leader, the SpTrf proteins are secreted into the coelomic fluid (CF), which was verified experimentally (38, 41).

Many versions of native (nat)SpTrf proteins with sufficient numbers of histidines in their histidine-rich region (**Supplementary Figures S1**, **S2**) can be isolated collectively by nickel affinity (42). They bind to Gram positive and negative bacteria and to yeast, and augment phagocytosis when bound to *Vibrio diazotrophicus* (38). However, nickel affinity does not allow differential isolation of individual natSpTrf proteins. Therefore, to understand the functions of individual SpTrf proteins, rather than all SpTrf proteins functioning together, rSpTrf-E1-Ec was investigated and found to have multiple binding targets. It binds LPS, flagellin, phosphatidic acid, β-1,3-glucan, *Vibrio diazotrophicus*, and *Saccharomyces cerevisiae*, however, it does not bind to peptidoglycan or *Bacillus* spp (35, 40). Upon the addition of LPS or phosphatidic acid, among other negatively charged molecules, rSpTrf-E1-Ec undergoes a structural transformation from disordered to mostly alpha helical (36, 40). Based on this unexpected characteristic, the proteins were re-named from the original of Sp185/333 to SpTransformer (SpTrf) with a concurrent renaming of the genes and messages. We have speculated that the sequence diversity among the natSpTrf proteins confers different multitasking functions that may overlap and provide maximal protection for sea urchins against pathogens (35), reviewed in (24)). Many different versions of the SpTrf proteins are expressed in response to immune challenge (43, 44) and may function optimally when secreted together into the CF. Surprisingly, rSpTrf-E1-Ec binds to *Vibrio diazotrophicus* but does not enhance phagocytosis of the bacteria by sea urchin phagocytes (38). Consequently, we proposed that the natSpTrf proteins with an E1 element pattern may function with other versions of natSpTrf proteins to drive phagocytosis of foreign and invading cells in *S. purpuratus*.

Previous work has shown that *E. coli* fails to express SpTrf proteins, with the exception of rSpTrf-E1-Ec (35). Consequently, to address the hypothesis of functional diversity among the SpTrf proteins, six were expressed in recombinant form through a eukaryotic expression system using Sf9 insect cells, *Spodoptera frugiperda* (45). Proteins were chosen based on differences in their element patterns (**Figure 1**) and gene expression levels in sea urchins (30, 33). Each of the six rSpTrf proteins are larger than expected relative to amino acid sequences deduced from the cDNAs, and all are glycosylated with N-linked oligosaccharides. They are stable compared to the non-glycosylated rSpTrf-E1-Ec, although one dimerized over time without loss of function. When cross-linked to inert beads (rSpTrf::beads), a subset of the proteins, including the dimerized version, augments phagocytosis by a subset of sea urchin phagocytes. Phagocytosis of rSpTrf::beads is enhanced by the polygonal and small phagocytes, whereas the discoidal phagocytes show low levels of base-line, constitutive phagocytosis. Our findings indicate that the polygonal and small phagocytes recognize and bind distinct rSpTrf proteins. This infers that at least some of the natSpTrf proteins secreted into the CF act as opsonins in the sea urchin immune system and that the polygonal phagocytes are the major cellular responders to targets opsonized by the natSpTrf proteins. This highlights the crucial functions of the natSpTrf proteins in the detection and elimination of microbes and other foreign particles from the CF and internal tissues and organs.

## 2 Materials and methods

### 2.1 Expression and isolation of rSpTrf proteins

Recombinant protein expression in Sf9 cells and isolation by nickel affinity was based on the method of Hossainey et al. (46, 47). Coding sequences from cDNAs encoding six SpTrf proteins (**Figure 1**; **Table 1**) were used to produce expression constructs with the *pMIB/V5-His* expression vector (ThermoFisher) by Gibson assembly (Gibson Assembly Cloning Kit, New England Biolabs). Coding sequence in the *pMIB/V5-His* vector adds a V5 tag and six histidines to the C terminus of the recombinant proteins. cDNA inserts and *pMIB/V5-His* were amplified with Gibson primers (**Supplementary Table S2**). The leader sequences in the deduced SpTrf proteins were identified by Signal P (ver 5.0; https://services.healthtech.dtu.dk/service.php?SignalP-5.0) and omitted from the amplicons as in Lun et al. (35). The truncated version of SpTrf-E2 was generated with a primer that inserted an early stop codon to result in rSpTrf-E2.1 (**Supplementary Figure S2**, **Supplementary Table S1**). Gibson assemblies were transfected into *E. coli* (Top 10 chemically competent, Invitrogen), selected with ampicillin, and the expected insert sizes of transfectants were confirmed by PCR. The cDNA encoding SpTrf-E2 was also used in standard vector construction by amplifying the insert using primers with *Hind*III and *Xho*I sequences (**Supplementary Table S2**) followed by restriction digests and ligation into the multiple cloning site in digested *pMIB/V5-His*. Selected constructs were sequenced across the ligation regions (GeneWiz/Azenta) to verify that the reading frame was maintained. Constructs with open reading frames for each of the rSpTrf proteins were transfected into Sf9 insect cells using cellfectin II (ThermoFisher) according to manufacturer’s instructions. Insect cell cultures were grown in Sf 900 II serum-free medium (Invitrogen) with gentamycin (15 µg/ml, ThermoFisher), and transfected cells were selected with blasticidin (10 µg/ml; Invitrogen). Preliminary protein expression and secretion into the culture medium was confirmed by Western Blot (see below) using rabbit-anti-V5-HRP antibody (Invitrogen). The culture was scaled up to 500 ml, the cells were pelleted, the culture medium was loaded into snakeskin dialysis tubing (3.5 kDa MW cut-off, 35 mm diameter, ThermoFisher), concentrated against polyethylene glycol flakes (PEG, 8000 MW; ThermoFisher) at 4°C for up to 8 hr or to about 85-120 ml, followed by dialysis against phosphate buffer (150 mM NaPO_4_ pH 8 or PBS pH 8) at 4°C for 16 to 48 hrs. The concentrated culture medium was spun at 600 x *g* to pellet aggregates and the supernatant was diluted with an equal volume of binding/wash buffer (500 mM NaCl, 50 mM NaPO_4_, 20 - 40 mM imidazole, pH 8) and incubated with 1.5 ml of washed Ni-NTA agarose beads (Qiagen) for 1 hr at room temperature (rt) with gentle rocking or rotation. Beads were pelleted at 60 x *g*, loaded into a 5 ml column, and washed 3 to 7 times with 5-7 ml of binding/wash buffer. rSpTrf proteins were eluted 5 to 7 times with 1 ml of elution buffer (binding/wash buffer with 250 mM imidazole), which were combined and loaded into snakeskin dialysis tubing, concentrated to ~1 ml on dry PEG, and dialyzed against PBS or phosphate buffer (pH 7.4) for 24 to 48 hrs at 4°C. The concentrated proteins were aliquoted and stored at -80°C. HALT (1%; Life Technologies) protease inhibitor was added to some samples. rSpTrf protein concentration was evaluated by OD^205^ on a spectrophotometer (Nanodrop 2000c [ThermoFisher] or NanodropOne [Thermo Scientific]) according to (36). The Scopes method (48) was used because the proteins do not have sufficient numbers of tryptophans, tyrosines, or cysteines in their sequences to be detected at OD^280^.

**Table 1:** rSpTrf protein sizes and predicted sites for post-translational modifications.

rSpTrf protein	Deduced size (kDa)^1^	Observed size range (kDa)^2^	Number of bands (WB)	Conserved sites for oligosaccharides^3^		PNGaseF treatment (kDa)^5^	cDNA Accession number	cDNA clone^6^	cDNA insert size (nt)
				N-linked^4^	O-linked				
01	29.85	43 – 50	2	4	17	43 to 38 50 to 44	EF065909	8-2441	826
E1	36.45	45 – 60	1	7	18	60 to 50	DQ183168	Sp0032	1119
E2-3	32.14	45 – 50	2	5	13	45 to 34 50 to 43	EF065832	2-2439	934
E2-4	32.14	45 – 80	2	5	13	80 to 70			
C1	42.35	55 – 70	1	7	15	70 to 60	EF066287	2-1514	1151
D1	41.72	45 – 60	1	7	20	55 to 45	EF066028	9-1542	1135
A6	54.63	65 – 75	1	7	18	75 to 70	EF065991	9-1525	1474

^1^Size prediction is based on the cDNA sequence that was amplified and used to generate the *pMIB/V5-HisA* expression construct. The deduced amino acid sequence was evaluated with the ExPASy MW calculator tool (https://web.expasy.org/compute_pi/).

^2^Protein size variation is based on multiple Western blot analyses over time.

^3^The number of sites predicted for N-linked and O-linked oligosaccharide addition is based on searches of the amino acid sequences with https://services.healthtech.dtu.dk/services/NetNGlyc-1.0 and https://services.healthtech.dtu.dk/services/NetOGlyc-4.0/, respectively.

^4^See also **Supplementary File 1**, **Supplementary Table S1**.

^5^Change in protein size from deglycosylation of N-linked oligosaccharides.

^6^cDNA sequences are reported in Terwilliger et al. (30, 33).

### 2.2 Western blots

rSpTrf proteins (1-2 µg) were separated by 8-12% SDS PAGE and transferred to PVDF membranes (Immobilon-P^seq^, EMD Millipore) for 10 min in a Trans-blot Turbo transfer system (BioRad). Filters were rinsed in Tris NaCl (TN, 25 mM Tris pH 7.4, 0.5 M NaCl) and blocked in blotto (5% milk in TN with 0.1% Tween 20 (TNT)) with rocking for 1.5 hrs at rt or overnight at 4°C. rSpTrf proteins were detected with rabbit-anti-V5-HRP (1500X to 3000X dilution in blotto; ThermoFisher). natSpTrf proteins on Western blots were detected with three rabbit-anti-natSpTrf-66, -68, -71 (3000 dilution each in blotto) that recognized different regions of the SpTrf proteins (**Supplementary Figure S2**), followed by goat-anti-rabbit-Ig-HRP (3000X dilution in blotto, Abcam) with rocking for 1 hour at rt (37). Filters were washed twice in TNT and twice in TN and incubated in ECL (Super Signal West Pico PLUS, ThermoScientific) and evaluated for chemiluminescence in a ChemiDoc Touch Imaging System (BioRad).

### 2.3 Deglycosylation

Conserved sites for N-linked and O-linked oligosaccharides in the sequences of the rSpTrf proteins were predicted using the on-line tools, https://services.healthtech.dtu.dk/services/NetNGlyc-1.0 and https://services.healthtech.dtu.dk/services/NetOGlyc-4.0/. Each rSpTrf protein (1-2 µg) or cell free CF (cfCF, 10-15 µl) was incubated with PNGaseF (1 µl, 0.5 U), *O*-glycosidase (2 µl, 8X10^4^ U), or *O*-glycosidase plus neuraminidase (2 µl, 40 U) at 37°C for 30 min, 75°C for 10 min, and chilled to 4°C according to the manufacturer’s protocol (New England Biolabs). Protein sizes were evaluated by Western blot as described above. Control proteins were incubated without the glycosidases.

### 2.4 Isolation of natSpTrf proteins by Ni affinity

CF was collected from 12 to 15 sea urchins in calcium and magnesium free sea water with EDTA and HEPES buffer [CMFSW-EH; 460 mM NaCl, 10.7 mM KCl, 7.04 mM Na_2_SO_4_, 2.38 mM NaHCO_3_, 70 mM EDTA, 20 mM HEPES pH 7.4 (44, 49)] and the cells were pelleted. The supernatant, or cfCF, was aliquoted and stored at -80°C until used. cfCF (35 ml) was incubated with washed Ni-NTA beads (1.75 ml; Invitrogen) by rotation at rt for 1 hr. Beads were pelleted at 800 x *g*, loaded into a 5 ml polypropylene column (ThermoFisher) and washed 3 times with 7 ml binding/wash buffer. Bound proteins were eluted 7 times with 1 ml elution buffer (10 mM NaCl, 50 mM NaPO_4_, 300 mM imidazole) for each elution. Elutions were combined, loaded into snakeskin dialysis tubing or a Slide-a-Lyzer mini dialysis unit (10,000 MWCO; ThermoFisher), and concentrated against polyethylene glycol 8000 (ThermoFisher) for 1-2 hrs at 4°C followed by dialysis against PBS (pH 7.4) over night at 4°C. Protein concentration was evaluated at OD^205^ according to Scopes (48). The natSpTrf proteins eluted from the Ni affinity column and unbound proteins were evaluated by Western blot with rabbit anti-SpTrf antibodies, and goat-anti-rabbit-Ig-HRP as described above. The natSpTrf proteins were aliquoted and stored at -80°C until used.

### 2.5 rSpTrf proteins cross-linked to magnetic beads

Magnefy magnetic beads (1 µm, Bangs Laboratories) with COOH groups on the surface were washed 3 times in MiliQ water (1 ml) according to the manufacturer to remove contaminants in the storage buffer (0.05% NaN_3_ in either de-ionized water or 5 mM Tris, 150 mM NaCl, Bangs Laboratory) and to reduce bead aggregation. Washed beads were incubated with 1-ethyl-3-(3-dimethyl-aminopropyl) carbodiimide hydrochloride (EDAC; PolyLink Protein Coupling Kit; Polysciences) in 200 μL for 15 min on a rotator at rt, followed by two wash steps using 200 μL coupling buffer as recommended to reduce bead aggregation. Beads were resuspended into 200 μL coupling buffer with each of the rSpTrf proteins or BSA (0.5 µM in 100 μL) for a total volume of 300 μL and incubated on a rotator at rt for 1 hour. Storage buffer (500 μL) was added and incubated with rotation at rt for 5 min to fill any remaining active sites on the beads with BSA. The beads were washed twice in 200 μL storage buffer, resuspended in 500 μL storage buffer, and stored at 4°C until used. The level of bead aggregation and protein binding was assessed by microscopy using rabbit-anti-V5-549 antibodies against the V5 tag on the rSpTrf proteins cross-linked to the beads. All wash steps were carried out with a magnet stand to collect the beads prior to removing the wash buffers.

### 2.6 Sea urchins

Adult sea urchins, *Strongylocentrotus purpuratus*, were purchased from the Southern California Sea Urchin Company (Corona del Mar, CA) after collection from the coast of southern California near San Diego. Sea urchins were fed rehydrated kelp (WEL-PAC) and maintained as described (50, 51).

### 2.7 Phagocytosis

CF was withdrawn from sea urchins as described (52) with 23 gauge needles attached to 1 ml syringes pre-loaded with 400 µl of CMFSW-EH to block clotting. Cells were dispensed into a 1.5 ml tube on ice containing 500 µL of CMFSW-EH and counted in a TC20 cell counter (BioRad).

#### 2.7.1 rSpTrf::beads incubated with phagocytes spun onto slides

Coelomocytes (3.65 x 10^4^ or 7.3 x 10^4^ cells/mL) were centrifuged onto Shandon Superfrost Plus positively charged microscope slides (Thermo Scientific or Epredia) at 1000 x *g* for 5 min at 4°C using slide holder assemblies with dual cytology chimneys (18 mm diameter, Hettich Zentrifugen). The slides in the slide holder assemblies were incubated at 4°C for 5 min to allow the cells to spread, and then moved to 14°C for 10 min. The CMFSW-EH was replaced with coelomocyte culture medium [CCM; 0.5 M NaCl, 5 mM MgCl_2_, 1 mM EGTA, 20 mM HEPES, pH 7.4 (37, 53)] every 10 min to 33%, 66%, 100% CCM as recommended (54). Two pipettes were used to aspirate CMSFW-EH and add CCM to ensure that the cells bound to the slide did not dehydrate. The cells were incubated at 14°C for 30 min, followed by replacing the CCM with rSpTrf::beads in CCM (100 beads/cell) and incubated for 20 min at 14°C. CCM (1 mL) was added to dilute and wash unbound beads from the cells, followed immediately by dismantling the slide holder assemblies. The cells were fixed for further processing and analysis (see below).

#### 2.7.2 rSpTrf::beads blocked with anti-SpTrf antibodies

Beads cross-linked to rSpTrf-E1 or rSpTrf-E2-3 were blocked using a mixture of rabbit anti-natSpTrf-66, -68, and -71 [100X dilution (37)], or with normal rabbit serum (NRS; 100X dilution) as the controls. Deglycosylation of N-linked oligosaccharides of rSpTrf-E1::beads and rSpTrf-E2-3::beads was conducted as described above using PNGaseF (New England Biolabs). Deglycosylated rSpTrf-E1::beads and rSpTrf-E2-3::beads were blocked with anti-natSpTrf antibodies or NRS. Beads were first incubated in blocking buffer (BB-PBS; 3% bovine serum albumin [BSA], 3% normal goat serum [NGS] in PBS) for 45 min at rt with rotation followed by incubation with the antibodies or NRS in BB-PBS for 1 hr at rt with rotation. Beads were washed 3 times with PBS, resuspended in 300 μL of storage buffer (Bangs Laboratory) and stored at 4°C until used. Coelomocyte association with glycosylated and deglycosylated, blocked, and control rSpTrf-E1::beads and rSpTrf-E2-3::beads was assessed as described above. Briefly coelomocytes were spun onto slides, the buffer was changed to CCM, and the cells were incubated with beads for 20 min at 14°C. The cells were washed and fixed for further processing and analysis (see below).

#### 2.7.3 rSpTrf::beads incubated with phagocytes in suspension

Beads cross-linked with proteins were washed with 200 μL artificial coelomic fluid [aCF; 10 mM CaCl_2_, 14 mM KCl, 50 mM MgCl_2_, 398 mM NaCl, 1.7 mM NaHCO_3_, 25 mM Na_2_SO_4_, pH 7.4 (30)] and resuspended in 100 μL of aCF. Coelomocytes in CMFSW-EH (~1 mL) were pelleted at 5000 x *g* for 7 min at 4°C. The supernatant was discarded and the cells were resuspended in 400 μL of aCF using a 1 ml pipette in which the tip was cut 5 mm from the end using a sterile razor blade to increase the bore diameter and reduce shear stress while the pelleted cells were resuspended. Cells were counted on the TC20 (Bio-Rad) cell counter and 1 x 10^5^ or 2 x 10^5^ cells/ml were transferred to a 1.5 ml tube. The rSpTrf::beads in aCF were added to the cells and the volume was brought to 500 μL with aCF (50 beads/cell). The cells were incubated with beads for 40 min at 14°C with agitation every 10 min. The cells and beads were moved to a dual cytology chimney in a slide holder assembly with a Superfrost Plus Slide (Epredia) and incubated for 20 min at 14°C to allow cells to settle and adhere to the slide. Chimneys were centrifuged in a swinging bucket rotor at 18 x *g* for 10 min at 4°C. The slide holder assembly was dismantled, and the slides were transferred to a cold metal sheet on ice. Live cells were blocked on ice for 1 hr in 125 μL of blocking buffer in aCF (BB-aCF; 3% BSA, 3% NGS). BB-aCF was tipped off the slides and cells that had been incubated with rSpTrf::beads were incubated for 1 hr with rabbit-anti-V5-549 (1000X dilution in BB-aCF; Rockland) at 0°C. Cells that had been incubated with BSA::beads were incubated in parallel with rabbit-anti-BSA (5,000X dilution in BB-aCF; ThermoFisher). Slides were transferred into a Coplin jar containing ice cold aCF and incubated for 5 min to remove unbound antibodies. Cells were fixed, permeabilized, and processed as described below and incubated with mouse-anti-actin (1500X dilution; MP Biomedicals) followed by goat-anti-mouse-488 (6000X dilution; Invitrogen), and 4',6-diamidino-2-phenylindole (DAPI). Concurrently, cells treated with BSA::beads were also incubated with goat-anti-rabbit-555 (5000X dilution; Invitrogen). Manual counts of beads per cell were done blind with regard to which protein was cross-linked to the beads.

### 2.8 Phagocyte fixation, permeabilization, and immunofluorescence

Phagocytes bound to slides were fixed as described previously (37, 52). Cells were incubated in prefix (0.000025% glutaraldehyde in CCM or aCF) for 5 min at 14°C, fixed immediately without washing (2% formaldehyde, 0.25% Triton X-100) in AC320 buffer (320 mM sucrose, 75 mM KCl, 2 mM MgCl_2_, 20 mM EGTA, 20 mM Pipes pH 7.4 (37, 53)) for 5 min at 14°C. Cells were permeabilized with ice cold methanol in a Coplin jar for 5 min at -20°C followed by a wash in PBS in a Coplin jar for 5 min at rt. Slides were moved to a humid chamber and cells were incubated with blocking buffer (BB-PBS; 3% BSA, 3% NGS in PBS) for 1 hr at rt followed by 3 washes in PBS for 5 min in a Coplin jar. To evaluate phagocytosis of beads by phagocytes spun onto slides, cells were incubated with rabbit-anti-V5-549 (3000X dilution in BB-PBS; Rockland) and mouse-anti-actin (1500X dilution in BB-PBS, MP Biomedicals) for 1 hr in a humid chamber followed by 3 washes in PBS of 5 min each in a Coplin jar. In parallel experiments to evaluate surface binding vs. phagocytosis of beads by phagocytes in suspension, cells were incubated with rabbit-anti-V5-549 at 0°C prior to fixation and were incubated with mouse anti actin after fixation and permeabilization, as described above. In the last step for all cytology protocols, cells were incubated with goat-anti-mouse-Ig-488 (6000X dilution in BB-PBS, Invitrogen) for 1 hr in a humid chamber. Cells were washed in PBS, and 10 μL of ProLong Gold antifade reagent with DAPI (Invitrogen) was added and a coverslip was added and sealed with clear nail polish. Cells were evaluated by fluorescence (Axioskop; Zeiss) and confocal (LSM 710; Zeiss) microscopy to quantify beads bound to the surface, beads phagocytosed, and the type of phagocyte was recorded.

### 2.9 Evaluation and statistics of phagocytes with beads

The data were visualized using boxplots to display both the mean and variability within each treatment group, to enable comparison between different experimental conditions. All visualizations and statistical analyses were conducted in R (version 4.3.0).

The association of beads per phagocyte for spun cells was determined by visual inspection using fluorescence microscopy (Axioskop; Zeiss). For each slide, 500 phagocytes per animal were evaluated, and cells from a minimum of six different sea urchins were used to evaluate each of the different proteins cross-linked to beads. The average number of beads associated with cells, and the percentage of phagocytes associated with at least one bead were evaluated. A Tukey ANOVA test was used to assess significant differences in the average number of phagocytes associated with beads cross-linked to different proteins.

Surface binding and phagocytosis of beads per phagocyte incubated in suspension, in addition to the type of phagocyte (polygonal, discoidal, or small) with beads was evaluated based on visual inspection by confocal microscopy (LSM 710; Zeiss). A minimum of 100 phagocytes per sea urchin were counted, and cells from a minimum of six sea urchins were evaluated for each protein. A Tukey ANOVA test was used to assess whether the average number of surface bound, and phagocytosed beads were significantly different among the different rSpTrf::beads. BSA::beads were used as the negative control. Data were also evaluated with a Dunnet test to identify significant differences between each of the rSpTrf::beads (treatment group) and the BSA::beads (control group). A two tailed paired *t*-test was used to evaluate significance between surface bound beads and phagocytosed beads for each protein cross-linked to beads.

### 2.10 Scanning electron microscopy

Based on methods described above, coelomocytes (10^5^ cells) were either i) spun onto a circular cover slip (18 mm; Fisherbrand item # 12-546) and incubated with rSpTrf-E2-3::beads in aCF, or ii) incubated with rSpTrf-E2-3::beads in solution in aCF followed by spinning onto the circular cover slips. Cells centrifuged onto the coverslips used the dual cytology chimneys with the same diameter as the coverslip. For both methods coelomocytes were incubated with 100 beads per cell for 1 hr followed by fixation for SEM. Coelomocytes were incubated in prefix solution (0.001% glutaraldehyde in aCF (52)) for 5 min at 14°C followed by the fixative solution (2.5% glutaraldehyde, 1% paraformaldehyde, in AC320) for 30 min at rt. Cover slips were washed 3 times with 0.12 mM sodium cacodylate buffer pH 7.4 (Electron Microscopy Sciences) for 10 min with shaking. The coelomocytes were transferred to the post-fixation solution (1% OsO_4_, 0.12 mM sodium cacodylate buffer, pH 7.4) for 1 hr at rt with shaking followed by 4 washes with deionized (DI) water for 10 min at rt. Dehydration was achieved by sequential immersion in ethanol at increasing concentrations (15%, 35%, 50%, 70%, 80%, 95%, 100%) for 10 min each at rt with shaking. Coverslips were submerged in hexamethyldisilane (HMDS) for 15 min at rt, moved into fresh HMDS, and incubated at rt overnight allowing for HMDS evaporation. Coelomocytes were sputter coated (Cressington 208HR Sputter Coater) with 2 nm PtPd and imaged with a scanning electron microscope (FEI Teneo LV SEM).

## 3 Results

### 3.1 rSpTrf proteins are expressed by Sf9 insect cells

**Figure 2:**
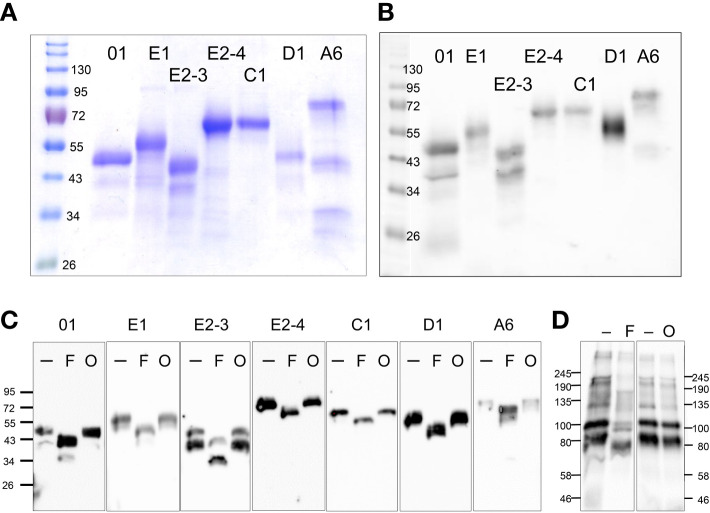
rSpTrf proteins are expressed in Sf9 insect cell cultures. **(A)** A Coomassie stained protein gel shows the rSpTrf proteins isolated from insect cells. **(B)** A Western blot shows the rSpTrf protein bands are based on recognition by rabbit-anti-V5-HRP. Broad Range protein standards (BioRad) are shown on the left. **(C)** The rSpTrf proteins are glycosylated with N-linked oligosaccharides. Each of the proteins was incubated with PNGaseF (F) to remove N-linked oligosaccharides, and a combination of neuraminidase and *O*-glycosidase (O) to remove O-linked oligosaccharides. Size changes were identified based on comparison to untreated controls (-). **(D)** CF containing natSpTrf proteins was treated with the glycosidases, which shows size changes with PNGaseF (F), but not with neuraminidase and *O*-glycosidase (O), compared to untreated controls (-). Rabbit-anti-natSpTrf antibodies (37) identify the natSpTrf proteins in the CF.

Our prior attempts to express rSpTrf proteins in *E. coli* culminated in a single version, rSpTrf-E1-Ec (originally called Sp0032), that was expressed and isolated successfully (35). Other rSpTrf expression constructs failed presumably because the proteins were lethal to *E. coli*. Although our previous studies provided information about the functions of rSpTrf-E1-Ec, questions remained regarding the functions of other versions, which were proposed to be different and perhaps complementary (35). Because of the toxicity of expressing rSpTrf proteins in *E. coli*, eukaryote cells were chosen for recombinant protein expression. Six distinct versions of SpTrf proteins were expressed in Sf9 insect cells that were selected based on size, sequence diversity, expression level (30) (**Figure 1**), as well as to compare the functions of rSpTrf-E1 to rSpTrf-E1-Ec. The choice of proteins included rSpTrf-01, -E1, -C1, -D1, and -A6 that were based on element pattern, in addition to rSpTrf-E2 and a variant called rSpTrf-E2.1, which show the highest expression level in coelomocytes (30). The rSpTrf proteins isolated from the cell cultures were initially evaluated by protein gels and Western blots to determine the molecular weights and expression levels, which showed that six constructs yielded recombinant proteins (**Figures 2A, B**). However, rSpTrf-E2.1, a truncated version of rSpTrf-E2, was not produced and secreted by the Sf9 cells even though the expression vector was incorporated into the genome of the cells and was transcribed (**Supplementary Text File 1**). Consequently, rSpTrf-E2.1 was not investigated further.

The six rSpTrf proteins ranged in molecular weight upon isolation from ~40 kDa to ~65 kDa (**Figures 2A, B**). However, two different isolations of rSpTrf-E2 resulted in proteins of different molecular weights (**Table 1**). A preliminary isolation of rSpTrf-E2 (denoted as rSpTrf-E2-4) was ~45 kDa, which was smaller than rSpTrf-A6, -D1, and -C1 (**Supplementary Figure S3A**). However, after a second large-scale isolation and storage, rSpTrf-E2-4 appeared on Western blots at ~80 kDa (**Table 1**; **Supplementary Figure S3B**), suggesting dimerization. In a different large-scale isolation of rSpTrf-E2 (denoted as rSpTrf-E2-3), it remained at 45 to 50 kDa over time (**Table 1**). Because the expression constructs for rSpTrf-E2-3 and rSpTrf-E2-4 included the identical SpTrf sequence, although with different ligation methods, the amino acid sequences of these two rSpTrf-E2 isolates were essentially identical. Although we cannot speculate as to why rSpTrf-E2-4 dimerized while rSpTrf-E2-3 did not, this difference provided an opportunity to test the functions of a dimerized compared to a non-dimerized version. This was particularly noteworthy given that dimerized rSpTrf-E1-Ec is not functional compared to the monomer (40).

When CF is collected from any sea urchin species, the native Trf proteins are mostly multimerized with only a few proteins within the expected size range of monomers (25, 26, 37, 43). To determine whether the rSpTrf proteins would multimerize with each other, rSpTrf-E2-3 and rSpTrf-E1 were mixed in equal mass with each of the other proteins and evaluated for multimerization based on size change on Western blots. However, results showed that the protein mixtures did not show increased band sizes on Western blots suggesting that multimers did not form (**Supplementary Figure S4**).

### 3.2 All of the rSpTrf proteins are glycosylated

The molecular weight of rSpTrf-E1-Ec deduced from Western blots matches the molecular weight deduced from the cDNA sequence (35). However, all of the rSpTrf proteins isolated from Sf9 cells were larger than predicted (**Figures 2A, B**; **Table 1**). Evaluation of the amino acid sequences of each rSpTrf protein predicted that each had numerous conserved sequence motifs for post-translational glycosylation (**Table 1**; **Supplementary Table S1**) (30). The rSpTrf proteins were evaluated for both N-linked and O-linked glycosylation by incubation with deglycosidases followed by Western blots to evaluate size changes. When the rSpTrf proteins and natSpTrf proteins were incubated with peptide N-glycosidase F (PNGaseF), *O*-glycosidase alone, or *O*-glycosidase plus neuraminidase A, proteins only decreased in size after treatment with PNGaseF compared to untreated controls (**Figure 2C**). However, the reduced sizes did not match the deduced sizes based on gene and transcript sequences. The combination of *O*-glycosidase with or without neuraminidase A did not alter the sizes of the proteins even though conserved motifs for O-linked oligosaccharides have been predicted (**Figure 2C**) (30, 33). However, the Sf9 cells likely did not add complex O-linked oligosaccharides to the rSpTrf proteins (55). Similar results were obtained for natSpTrf proteins after treatment with *O*-glycosidase (**Figure 2D**). This suggested that the recombinant and native natSpTrf proteins were only glycosylated with N-linked oligosaccharides. The analysis did not address the possibility that the proteins may have had O-linked oligosaccharides that were not removed by the *O*-glycosidase treatment. Whether the increased molecular weight of the rSpTrf proteins was due to other post-translational modifications or associations with other unknown molecules was not addressed.

### 3.3 rSpTrf::beads show augmented association with phagocytes

**Figure 3:**
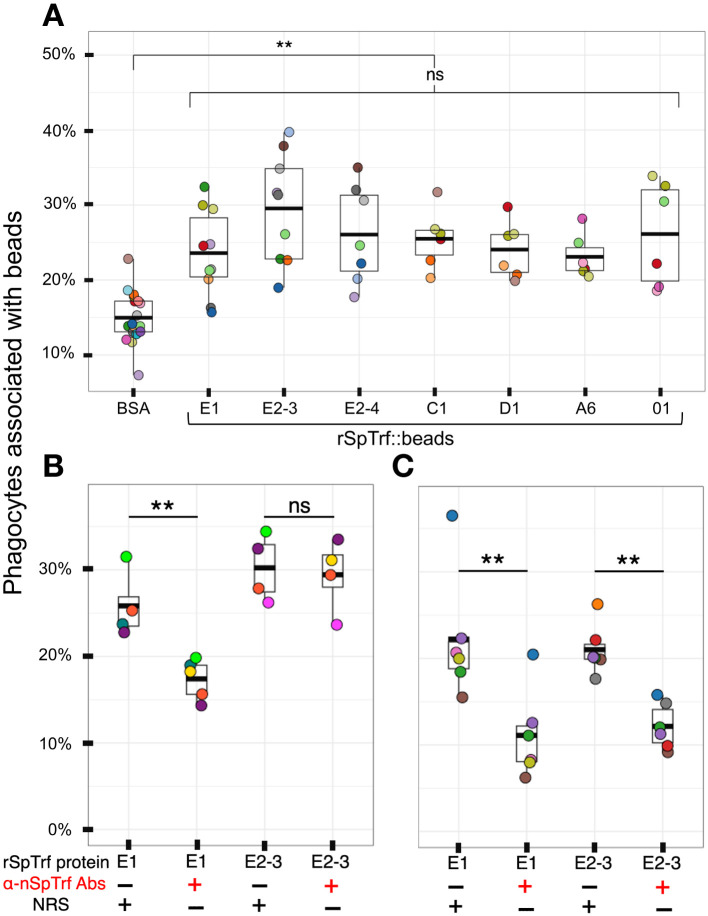
All rSpTrf proteins cross-linked to beads augment the association with phagocytes. **(A)** When phagocytes bound to slides are incubated with rSpTrf::beads, a greater percentage of phagocytes (**, *p* < 0.05) are associated with beads compared to the control BSA::beads. There are no significant differences (ns) among the rSpTrf proteins relative to the association of beads with phagocytes. **(B)** Pre-incubation of rSpTrf-E1::beads and rSpTrf-E2-3::beads with three rabbit-anti-natSpTrf antibodies reduces the percentage of phagocytes associated with rSpTrf-E1::beads (**, *p* < 0.05), but has no significant effect on rSpTrf-E2-3::beads (ns). **(C)** Deglycosylation of the rSpTrf::beads prior to blocking with rabbit-anti-natSpTrf antibodies decreases (**, *p* < 0.05) the percentage of phagocytes with associated rSpTrf-E1::beads and rSpTrf-E2-3::beads compared to beads that are not pre-incubated with the antibodies. Each dot represents the results for phagocytes collected from a single sea urchin. For each protein at least six animals were tested, and at least 500 cells per animal were counted for bead association. The protease inhibitor, HALT (1%), was added to all proteins. The box plots show the average and interquartile range of each bead treatment associated with phagocytes. A Tukey ANOVA test was used to evaluate the significance in bead association among cells incubated with the different rSpTrf proteins cross-linked to beads.

Previous analysis of rSpTrf-E1-Ec reported that it bound to *Vibrio diazotrophicus* in addition to *Vibrio* flagellin (35), however it did not augment phagocytosis of opsonized *V. diazotrophicus* by phagocytes (38). In comparison, natSpTrf proteins isolated from cfCF by nickel affinity opsonized *V. diazotrophicus* and augmented phagocytosis by phagocytes. Consequently, it was postulated that the natSpTrf-E1 isoform may interact with other versions during the opsonization and phagocytosis of foreign particles and microbes and that other natSpTrf proteins had different but overlapping functions (35, 36, 38). Because the expression of other rSpTrf proteins from *E. coli* failed, this possibility was not addressed. With the expression of several rSpTrf proteins from insect cells, the hypothesis was tested using rSpTrf proteins bound to inert beads. The use of inert beads removed the confounding effects of surface PAMPS on bacteria, such as LPS, that are detected by sea urchin cells (56–60), induce the expression of the *SpTrf* gene family (30), and likely drive phagocytosis of Gram negative bacteria (38). The use of rSpTrf::beads limited the interactions between the cells and beads exclusively to the phagocyte cell surface and the rSpTrf proteins. Initial analyses of phagocytosis employed magnet capture of phagocytes that had taken up magnefy beads. However, results showed significant background using coelomocytes with this approach (**Supplementary Text File 2**). Consequently, opsonin function of rSpTrf proteins was inferred from phagocytosis of rSpTrf::beads by phagocytes spun onto slides followed by visual inspection using microscopy. The percentage of phagocytes associated with rSpTrf::beads was significantly (*p* < 0.05) higher compared to BSA::beads, which served as the negative control (**Figure 3A**).

Because there were no significant differences in the percentages of phagocytes associated with different rSpTrf::beads, this suggested that the phagocytes had comparable binding for the different rSpTrf proteins. However, these results did not verify specific interactions that occurred directly between the rSpTrf::beads and the phagocytes. To address this, three rabbit-anti-natSpTrf antibodies [see **Supplementary Figure S2** that shows the peptide sequences to which the antibodies were raised and their locations in the proteins; see also (37)] were incubated with rSpTrf-E1::beads and rSpTrf-E2-3::beads prior to mixing with the cells. rSpTrf-E1 and -E2-3 were chosen because they had the lowest and highest percent association with phagocytes, respectively (**Figure 3A**). This was done to determine whether the antibodies bound to the rSpTrf::beads would interfere with the association between the rSpTrf proteins and the phagocytes. Results showed a significant reduction in the association of rSpTrf-E1::beads with cells, suggesting a specific interaction between rSpTrf-E1 and the phagocytes (**Figure 3A**). However, the anti-SpTrf antibodies did not reduce the association between the rSpTrf-E2-3::beads and the phagocytes. To determine whether the N-linked oligosaccharides were blocking antibody access to one or more of the binding sides on the proteins, both rSpTrf-E1::beads and rSpTrf-E2-3::beads were deglycosylated with PNGaseF prior to repeating the experiment. Results showed that the anti-natSpTrf antibodies reduced the association of both deglycosylated rSpTrf-E1::beads and rSpTrf-E2-3::beads with phagocytes compared to the glycosylated proteins (**Figure 3C**). However, deglycosylation also decreased general bead association with the phagocytes when the antibodies were not used for blocking (compare **Figures 3B, C**). These findings suggested that the interactions between rSpTrf::beads and the phagocytes was specific rather than constitutive, non-specific binding to foreign particles. This specificity is in agreement with a similar result for PlTrf proteins in the sea urchin, *Paracentrotus lividus* (26).

### 3.4 Phagocytes spun onto slides are incapable of internalizing beads

**Figure 4:**
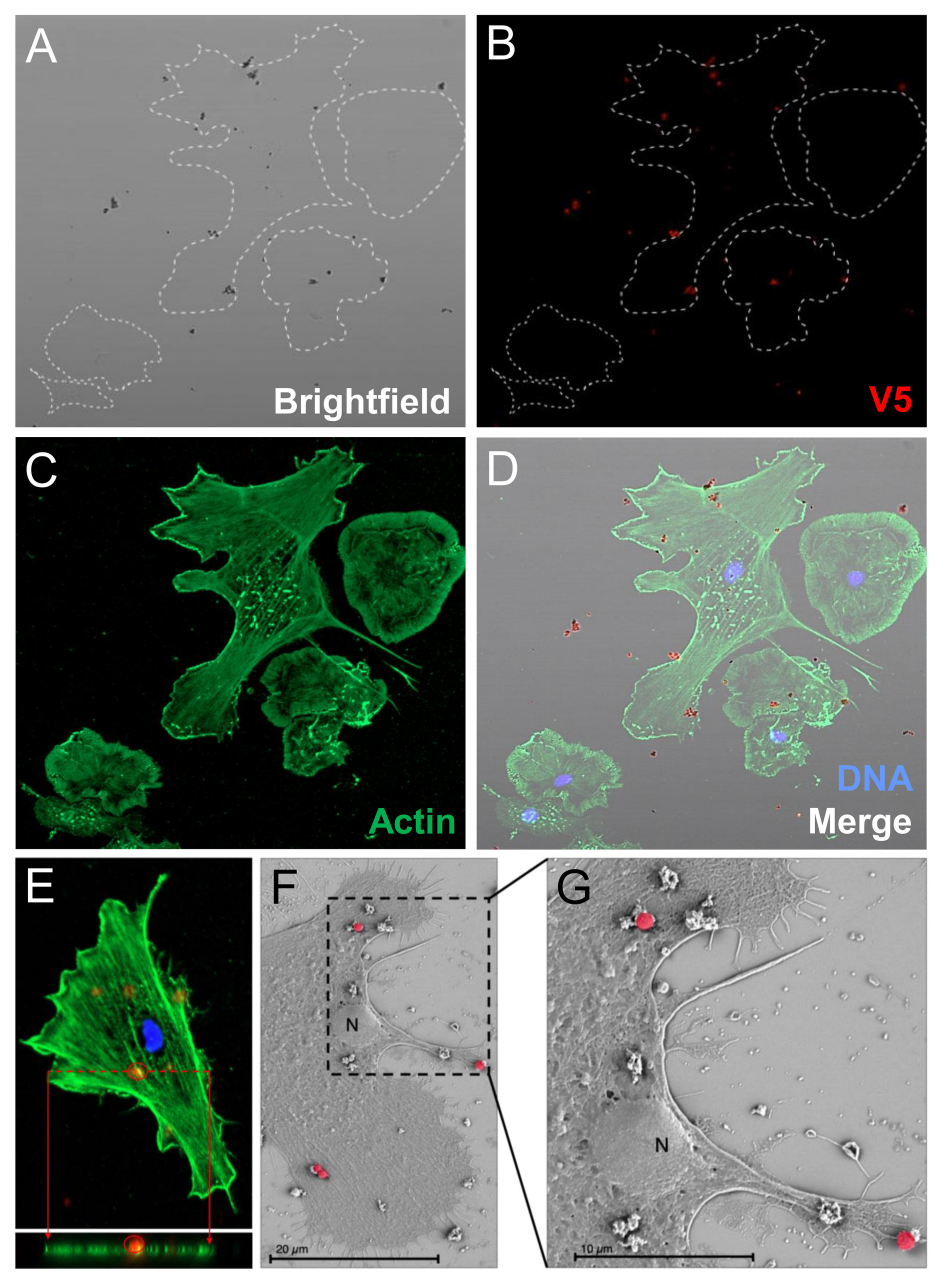
Phagocytes spun onto glass slides prior to incubation with rSpTrf::beads do not phagocytose surface bound beads. Spun cells with associated beads were incubated with rabbit-anti-V5-549 at 0°C followed by fixation and permeabilization for mouse-anti-actin and goat-anti-mouse-Ig-488 incubation. All beads were bound by rabbit-anti-V5-549 and appear red identifying only surface bound beads. No unlabeled beads are identified indicating that none are phagocytosed. **(A)** Bright field shows the location of all beads. **(B)** rSpTrf::beads are identified by rabbit-anti-V5-549 by UV epifluorescence. **(C)** Actin labeling of the cytoskeleton defines the types of phagocytes in the field; polygonal (P) and discoidal (D) phagocytes. **(D)** The merged image shows that all beads are labeled red indicating that no beads are internalized. **(E)** A Z-stack analysis by confocal microscopy shows that all beads are positioned above the cell surface and that none are positioned within the actin cytoskeleton. This indicates that the rSpTrf::beads are only surface bound. The DNA is labeled with DAPI. **(F, G)** Scanning electron microscopy confirms that spun phagocytes do not take up surface bound rSpTrf-E2-3::beads (false red color). Spun phagocytes are extremely flat, which decreases the vertical cytosolic space so that 1 µm beads are not internalized. The comparison between the beads bound to the cell surface vs. those bound to the slide surface suggests cellular recognition of rSpTrf-E2-3::beads.

Based on the initial evaluation by standard microcopy, it was not possible to discern whether the rSpTrf::beads had been phagocytosed or whether they were only bound to the surface of the cells. To test this, phagocytes spun on slides were incubated with rSpTrf::beads, then moved to 0°C and incubated with a rabbit-anti-V5-549 antibody before fixation and permeabilization. This limited the binding of the anti-V5-549 antibody to rSpTrf::beads that were on the surface of the phagocytes and any beads that had been phagocytosed were inaccessible by the antibody. This approach showed that all beads associated with the phagocytes had bound the antibody and appeared red suggesting that all were surface bound and none were internalized (**Figures 4A-D**). Z-stack evaluation by confocal microscopy confirmed that the rSpTrf::beads were not phagocytosed and that they were consistently located above the actin cytoskeleton, suggesting that they were surface bound (**Figure 4E**). The approach that blocked antibody penetration into the cytosol prior to fixation and permeabilization was confirmed with a mouse-anti-actin. The absence of cytoskeletal labeling indicated that the antibody was blocked from crossing the cell membrane (**Supplementary Figure S5**).

To further verify that beads were limited to the surface of phagocytes and not internalized, scanning electron microscopy was used to visualize the phagocyte interactions with rSpTrf-E2-3::beads. This rSpTrf protein was chosen because it showed the highest average percentage of cells with beads on the surface (**Figure 3A**). The SEM images suggested that spun cells were able to recognize the rSpTrf-E2-3::beads because the number of beads on the cell surface (n = 7; the cell was 22% of the area in the field of view) was generally higher than the number of beads on the glass slide (n = 5; 78% of the area) (**Figures 4F, G**; **Supplementary Figure S6**). However, the morphology of the spun cells suggested that they were spread too thinly to take up the 1 µm beads that were bound to the surface.

### 3.5 Phagocytes bind and internalize rSpTrf::beads when incubated in solution

**Figure 5:**
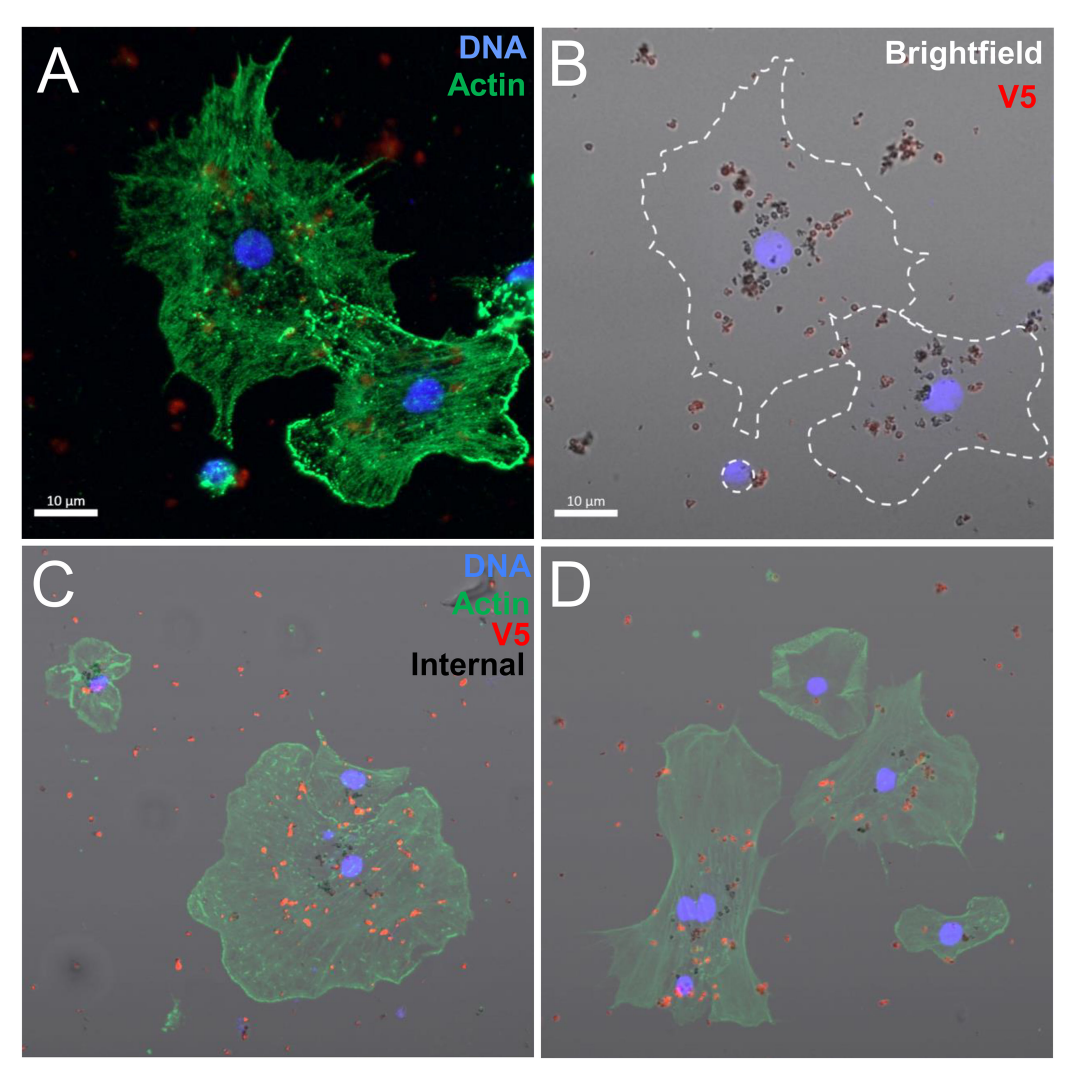
Coelomocytes incubated with rSpTrf-E2-3:beads in solution result in bead phagocytosis. When coelomocytes are incubated with beads in solution, they are capable of phagocytosis. Beads on the outside of the cells are labeled with rabbit-anti-V5-549 (red) before fixation and permeabilization, which blocks antibody access to the internalized beads (black). **(A, B)** Two polygonal phagocytes take up rSpTrf-E2-3::beads. The bright field image shows red beads that are located on the cell surface and unlabeled black beads that are phagocytosed. **(C, D)** Merged images show phagocytes with red beads on the surface and black beads that have been phagocytosed.

**Figure 6:**
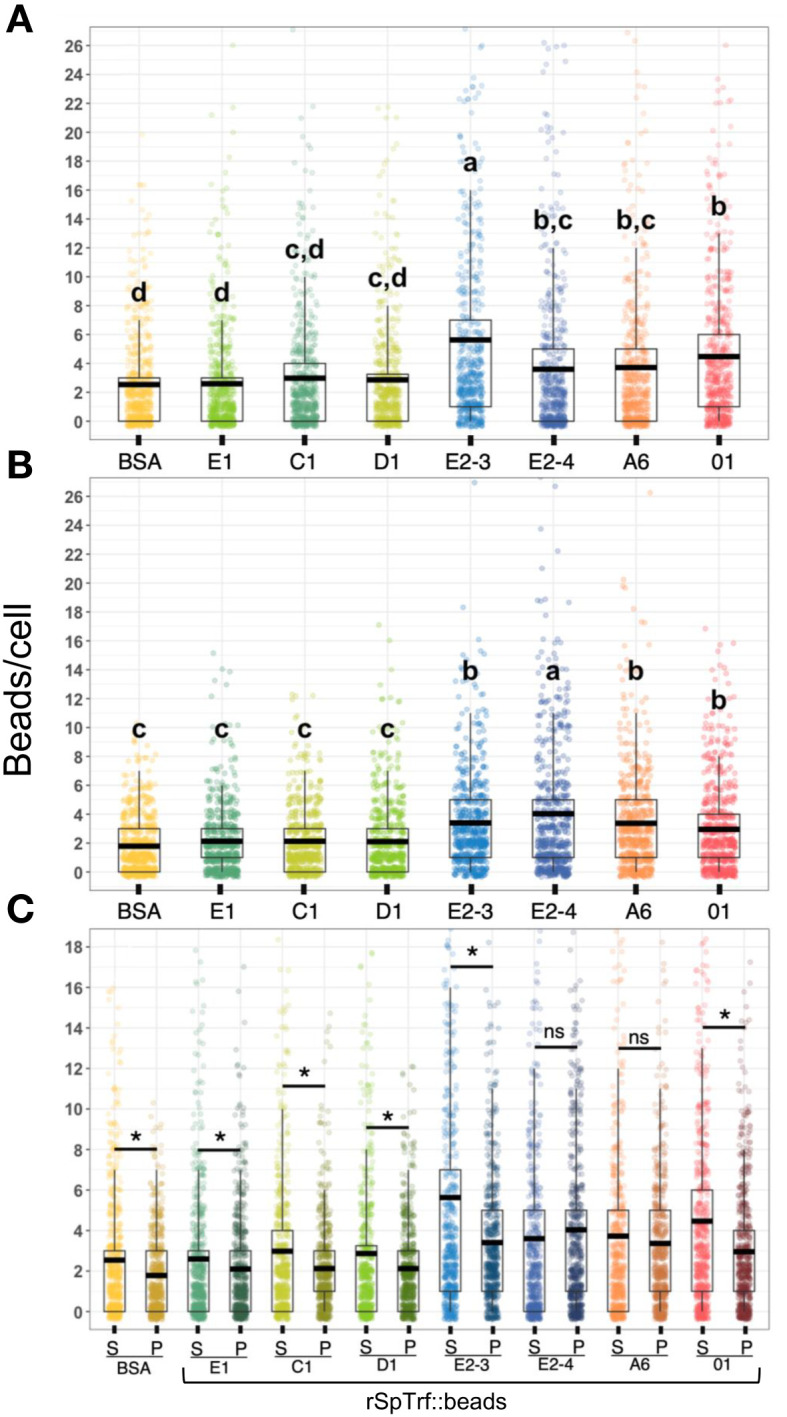
The rSpTrf::beads show significant differences in the number of beads bound and phagocytosed by phagocytes. The numbers of beads per cell were evaluated for phagocytes based on beads bound to the cell surface and beads phagocytosed. The box plots show the average and interquartile range of each rSpTrf::bead treatment associated with phagocytes. **(A)** Differences in the number of rSpTrf::beads bound to the cell surface is based on the rSpTrf protein. **(B)** Differences in the number of phagocytosed rSpTrf::beads by phagocytes resolves to those with specific phagocytosis and those that are not different from the control BSA::beads. Data analysis was based on a total of 600 cells counted for each rSpTrf::bead and cells collected from six different sea urchins. Statistical analysis employed Tukey ANOVA and significance was set to *p* < 0.05. The letters above the boxplots indicate significant differences or similarities among groups. See also **Table 2** for the different levels of binding and phagocytosis of the rSpTrf::beads based on statistical results using the Dunnet test. **(C)** The number of surface bound beads (S) compared to the number of phagocytosed beads (P) shows, for most proteins, that there are significantly more rSpTrf::beads bound to the cell surface than are phagocytosed (*, paired *t*-test, *p* < 0.05). There are no significant differences (ns) between surface bound and phagocytosed rSpTrf-E2-4::beads and rSpTrf-A6::beads.

To address the inability of spun and flattened phagocytes to take up the rSpTrf::beads, the approach for incubating the cells with the rSpTrf::beads was modified. Cells were incubated with the beads in solution, followed by a low-speed spin onto slides. As above, cells held at 0°C on slides were incubated with the rabbit-anti-V5 antibody prior to fixation and permeabilization to identify the beads on the surface vs. those that had been phagocytosed and were unavailable to the antibody. Results showed that in addition to binding beads on the surface, phagocytes also internalized the rSpTrf::beads when incubated in solution (**Figure 5**; **Supplementary Figure S7**). Different rSpTrf::beads showed differences in both surface binding and phagocytosis with variations in the number of beads per phagocyte (**Figure 6A**). There were more rSpTrf-E2-3::beads bound to the surface of cells, and results for the other rSpTrf::beads revealed several levels of binding to cell surfaces (**Table 2**). There were more phagocytosed rSpTrf-E2-4::beads per cell compared to the other rSpTrf::beads (**Figure 6B**). Furthermore, the numbers of phagocytosed beads per cell were slightly different than the number of surface beads per cell. These findings suggested that phagocytes could recognize rSpTrf-E2-3, -A6, -01 and -E2-4 cross-linked to beads, whereas binding and uptake of beads cross-linked to rSpTrf-E1, -C1, and -D1 were not different from the control BSA::beads (**Table 2**).

**Table 2:** Different types of phagocytes and different rSpTrf::beads show different levels of binding and phagocytosis^1^.

Cell type	Bead location	Level of binding and phagocytosis of rSpTrf::beads^2^			
		High	Medium	Low	ns
All phagocyte types	Surface Bound	E2-3, A6, 01	E2-4		E1, C1, D1
	Phagocytosed	E2-4, E2-3, A6, 01			E1, C1, D1
Polygonal	Surface Bound	E2-4, E2-3, A6, 01			E1, C1, D1
	Phagocytosed	E2-4, E2-3, A6, 01	E1	C1	D1
Discoidal	Surface Bound			01	E2-4, E2-3, A6, E1, C1, D1
	Phagocytosed			C1	E2-4, E2-3, A6, 01, E1, D1
Small	Surface Bound	E2-3			A6, 01, E2-4, E1, C1, D1
	Phagocytosed		E2-4	E2-3	A6, 01, E1, C1, D1

^1^These results are also shown in **Figures 7**, **8**, and as quantified in **Figure 9** in which the data are evaluated by the Tukey ANOVA test.

^2^Significant differences in the number of rSpTrf::beads per cell compared to BSA::beads is based on the Dunnet test. High, *p* ≤ 0.001; medium, *p* ≤ 0.01; low, *p* ≤ 0.05; ns, not significantly different from the control BSA::beads.

**Figure 7:**
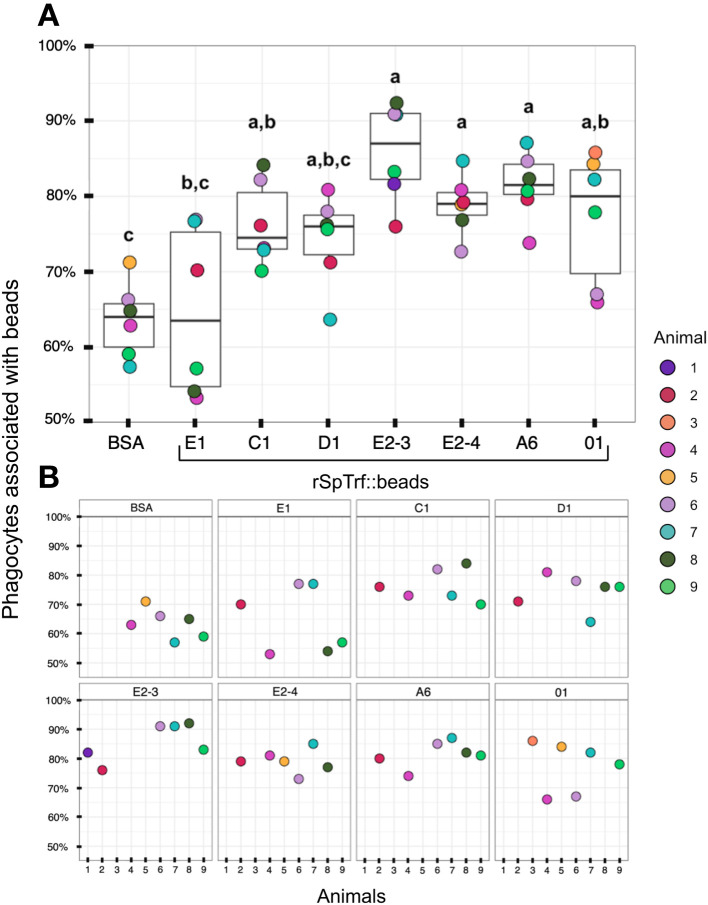
More phagocytes are associated with a subset of rSpTrf::beads compared to the control BSA::beads. Beads cross-linked to rSpTrf-E2-3, -E2-4, -A6, -C1, and -01 are associated with significantly more phagocytes (*p* < 0.05) than beads cross-linked with rSpTrf-D1, -E1, and the BSA control. **(A)** The box plots show the average and interquartile ranges for the average percentage of phagocytes with at least one bound or phagocytosed bead for the different rSpTrf::beads tested. Each dot represents the results for phagocytes collected from a single sea urchin. For each rSpTrf::bead, cells from at least six animals were tested, and at least 100 cells per animal were counted for bead association. Statistical analysis employed Tukey ANOVA and significance was set to *p* < 0.05. The letters above the boxplots indicate significant differences or similarities among groups. **(B)** The percentage of phagocytes with at least one bead shows variations among individual sea urchins. Cells from some animals were tested with subsets of rSpTrf::beads. Dots missing for individual animals indicate treatments that were not carried out for that animal.

When the results for beads per phagocyte were re-evaluated as the percentage of phagocytes associated with at least one bead, similar results were obtained relative to the rSpTrf protein cross-linked to the beads (**Figure 7A**). There were significantly more phagocytes associated with beads cross-linked to rSpTrf-E2-3, -E2-4, -A6, -01, and -C1 compared to beads cross-linked to rSpTrf-E1, -D1, and -BSA. However, the results for rSpTrf-C1::beads were different; there were significantly more cells associated with at least one rSpTrf-C1::bead compared to the number of C1::beads per phagocyte (compare **Figures 7A**, **6A, B**). When the number of rSpTrf::beads per cell on the surface was compared to those phagocytosed for the same rSpTrf protein, results generally showed significantly fewer beads inside the cells compared to those on the surface (**Figure 6C**). However, this did not apply to rSpTrf-E2-4::beads and rSpTrf-A6::beads, which showed similar numbers of beads bound to the surface and those phagocytosed. When cells collected from different sea urchins were evaluated, differences in the recognition of the same rSpTrf protein among the animals were observed (**Figure 7B**). The average number of phagocytes associated with rSpTrf::beads was variable and ranged from 53% (sea urchin 4 and rSp-Trf-E1) to 92% (sea urchin 8 and rSpTrf-E2-3). Although for cells from other sea urchins (*e.g.*, 6 and 7) similar numbers of phagocytes (75%) were associated with at least one bead cross-linked to rSpTrf-E1, -E2-4, and -01. Overall, these findings suggested that phagocytes showed specific recognition of certain rSpTrf proteins cross-linked to the beads. Some rSpTrf proteins enhanced bead binding and internalization by phagocytes, whereas others showed patterns similar to those of the control beads.

### 3.6 Polygonal phagocytes are the major phagocyte for recognition and uptake of rSpTrf::beads

**Figure 8:**
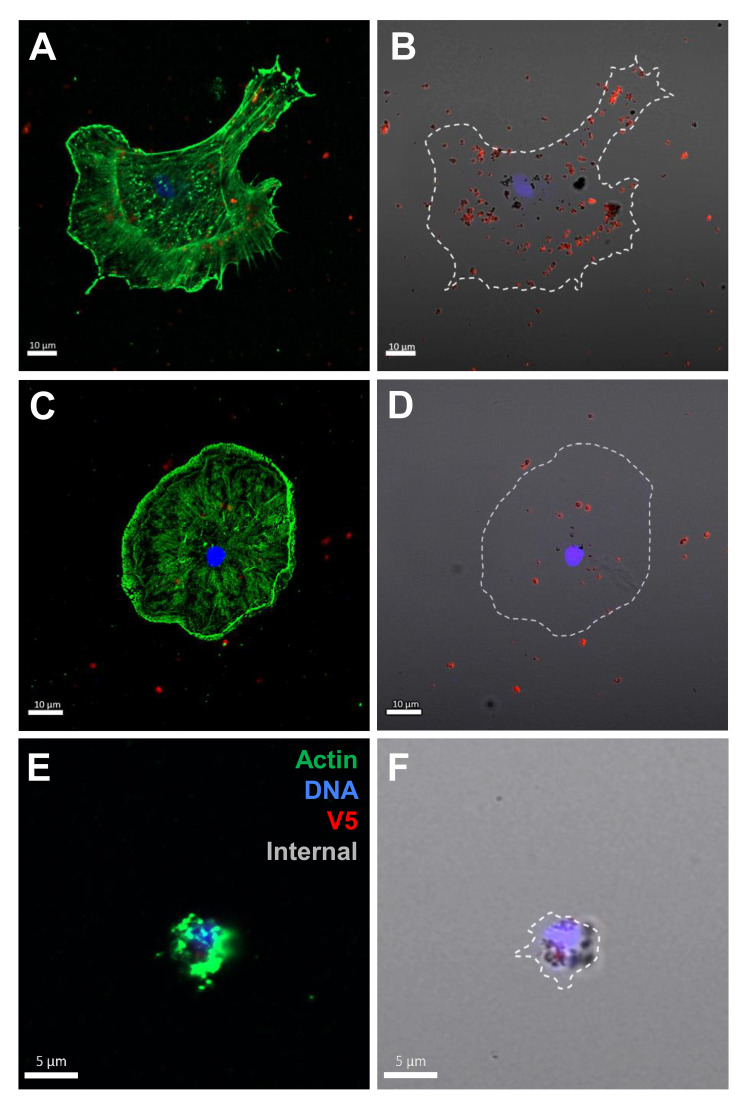
Polygonal, discoidal, and small phagocytes bind and phagocytose rSpTrf-E2-3::beads. Cells incubated with rabbit-anti-V5-549 prior to permeabilization labeled the beads bound to the cell surfaces, whereas internalized beads were not labeled because the antibodies were blocked from penetrating the plasma membrane. Identification of phagocyte type relied on actin cytoskeleton organization and cell size (37, 53). A polygonal cell **(A, B)** and a discoidal cell **(C, D)** have beads on the surface (red) as well as internalized beads (black). A small phagocyte **(E, F)** is also capable of bead phagocytosis showing unlabeled black beads around the nucleus.

**Figure 9:**
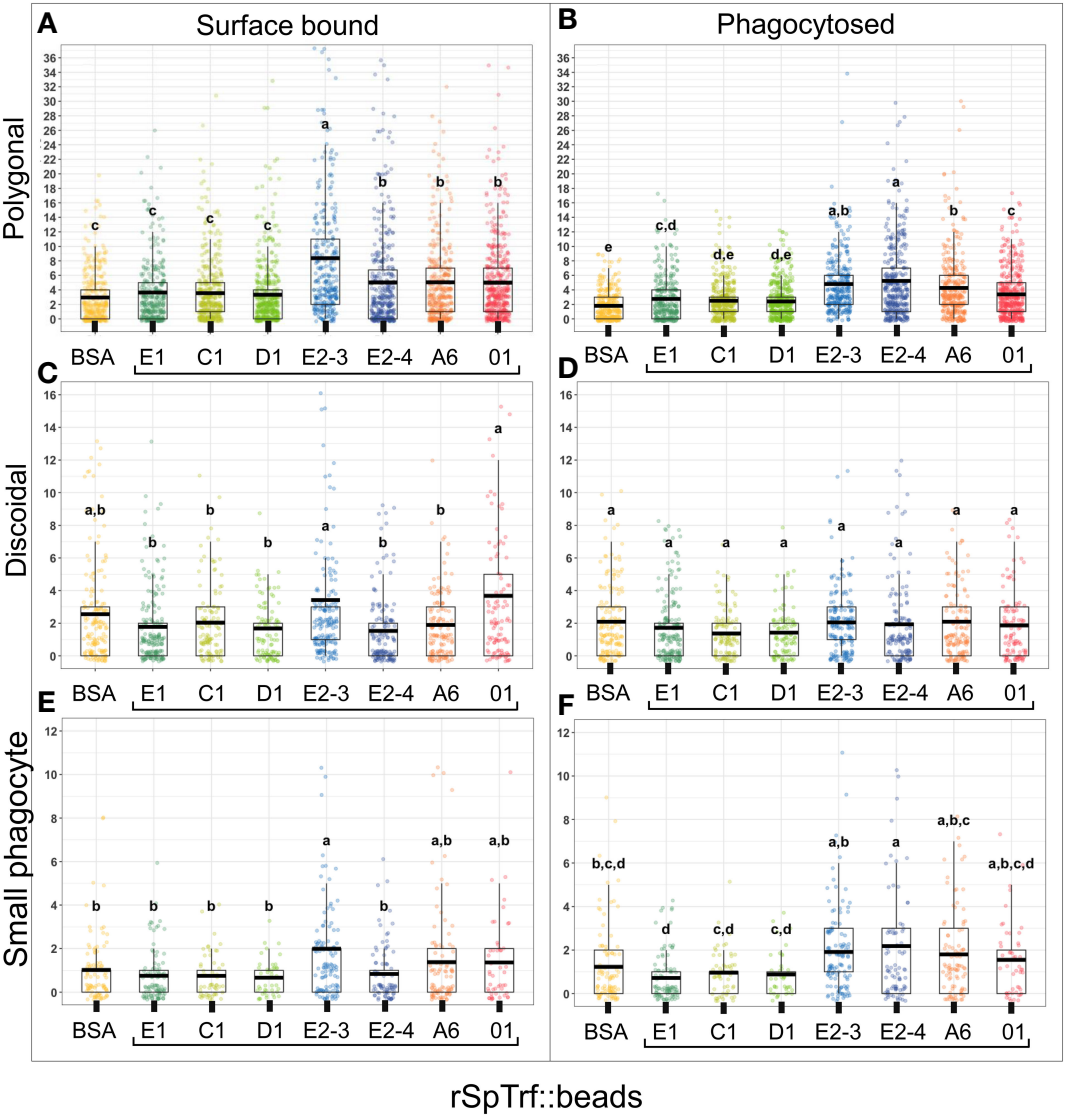
Different types of phagocytes show differences in binding and phagocytosis of rSpTrf::beads. Differential cell counts of cells processed for rabbit-anti-V5-459 labeling, as shown in **Figure 8**, are quantified for binding and phagocytosis of different rSpTrf::beads. The box plots show the average and interquartile range of each rSpTrf::bead treatment associated with phagocytes. Statistical analysis employed Tukey ANOVA and significance was set to *p* < 0.05. The letters above the boxplots indicate significant differences or similarities among groups. **(A, B)** The polygonal phagocytes show elevated levels of binding and phagocytosis per cell for a subset of the rSpTrf proteins compared to the other phagocyte types. They also show differential binding and phagocytosis based on the rSpTrf protein cross-linked to the beads. **(C, D)** The discoidal phagocytes bind and phagocytose fewer beads compared to the polygonal phagocytes, and do not differentiate among the rSpTrf::beads or the BSA::beads. **(E, F)** The small phagocytes do not bind or phagocytose many beads, likely due to their small size of about 5 µm, but they show differences in binding and phagocytosis depending on the rSpTrf cross-linked to the beads. Several levels of binding and phagocytosis for the different cell types and different rSpTrf::beads are shown in **Table 2**.

The coelomocytes of the phagocyte class of cells in the purple sea urchin have been characterized based on their numbers in the CF, their cytoskeletal morphology, and differences in size ((53, 61, 62); reviewed in (63, 64)). These colorless, filopodial or lamellipodial cells bind tightly to glass and are phagocytic (37, 65, 66), even though this has not been tested for small phagocytes (39). By discriminating polygonal, discoidal, and small phagocytes based on their distinct morphologies and sizes, these cell subsets were compared for surface binding and phagocytosis of the SpTrf::beads. Medium phagocytes were not identified and may only appear in response to loss of CF (39). Results showed that all three types of phagocytes bound and phagocytosed the rSpTrf::beads (**Figure 8**). The polygonal phagocytes associated with the most rSpTrf::beads and showed two levels of binding and phagocytosis of the several rSpTrf proteins cross-linked to the beads (**Figures 9A, B**; **Table 2**). There were significantly more rSpTrf-E2-3::beads bound to the cell surfaces, and an intermediate level of beads cross-linked with rSpTrf-E2-4, -A6, and -01 (**Figure 9A**). Surface binding of rSpTrf–E1, –C1, and –D1 were not different from the BSA::beads. Polygonal phagocytes internalized more rSpTrf-E2-4::beads (**Figure 9B**) with some differences in phagocytosis of the other rSpTrf::beads compared to those bound to the surface (**Table 2**). In general, the rSpTrf-E2::beads showed the greatest number of beads bound to the surface and internalized by polygonal phagocytes. In contrast, the discoidal cells bound and phagocytosed rSpTrf::beads (**Figures 8C, D**), but quantification indicated that the interactions were either not different from the BSA::beads (**Figures 9C, D**) or that there were low levels of binding or phagocytosis of rSpTrf-01 and -C1 cross-linked to beads (**Table 2**). Consequently, the discoidal cells demonstrated low levels of base-line, constitutive binding and phagocytosis of rSpTrf::beads without distinction among the rSpTrf proteins. It is noteworthy that while the discoidal phagocytes did not differentiate among the various rSpTrf::beads and the BSA::beads, they exhibited a comparable number of bound and phagocytosed BSA::beads as the polygonal phagocytes (compare **Figures 9A, B** with **C, D**). This suggested that the discoidal and polygonal phagocytes exhibited similar levels of constitutive phagocytosis.

Small phagocytes have been identified more recently (37, 50) than the large phagocytes and therefore little is known about their functions, including whether these cells are capable of phagocytosis. Imaging of small phagocytes illustrated their capabilities for binding and phagocytosis of rSpTrf::beads (**Figures 8E, F**). These small cells showed elevated binding for beads cross-linked with rSpTrf-E2-3, -A6, and -01, whereas the remaining rSpTrf::beads were not different from the BSA::beads (**Figure 9E**; **Table 2**). Small phagocytes internalized rSpTrf-E2-4::beads more readily than the other rSpTrf proteins (**Figure 9F**; **Table 2**). These results demonstrated that these small phagocytes were capable of taking up multiple beads. Overall, these findings showed that the three types of phagocytes functioned differently for binding and phagocytosis that ranged from specific associations with specific rSpTrf::beads to non-specific, constitutive phagocytosis. The polygonal phagocytes appeared as the major phagocytic cell in sea urchins.

## 4 Discussion

Expanded gene families encoding immune response proteins are present in a wide variety of invertebrates and are a basic attribute of innate immunity in these animals. Examples include the *C1q* gene family in the oyster, *Crassostrea gigas* (67, 68), the gene family encoding the fibrinogen related proteins (FREPs) in molluscs (69), the gene family encoding the variable chitin binding proteins (VCBPs) in amphioxus, *Branchiostoma floridae* (70), several gene families encoding AMPs in the housefly, *Musca domestica* (17), and the *SpTrf* gene family in euechinoids (24). The significant sequence diversity among the *SpTrf* genes, their expression patterns in response to immune challenge, and the functions of rSpTrf-E1-Ec for binding *Vibrio diazotrophicus* and PAMPs has established this system as an essential part of immune responses in sea urchins (reviewed in (24)). Here we extend these investigations to understand the functions of other rSpTrf proteins expressed by insect cell cultures. All of the rSpTrf::beads associated with significantly more phagocytes that were spun and flattened than control BSA::beads, suggesting that phagocytes have a cell surface mechanism for recognizing and binding the rSpTrf proteins. A subset of these proteins mediate binding of inert beads to phagocyte surfaces when cells are incubated in suspension, which enables them to phagocytose bound beads. Furthermore, the type of phagocyte that interacts with the bead shows variations in this process. The rSpTrf-E2 proteins show the highest level of binding to and phagocytosis by polygonal and small phagocytes. This is noteworthy because *SpTrf* genes encoding proteins with the E2 element pattern are the most highly expressed members of the gene family in response to immune challenges (29, 30) even though they are not the most numerous members of the family (31). However, gene expression level and binding function does not correlate for the other SpTrf proteins. The third highest expressed genes are those that encode SpTrf-C1, however these proteins do not show elevated binding functions (30). Alternatively, the *SpTrf-A* genes have very low expression, whereas the rSpTrf-A6 protein shows elevated binding. The rSpTrf proteins show several levels of binding and phagocytosis with some that do not support binding and phagocytosis above the constitutive level of the control BSA::beads. When these results are applied to the functions of the sea urchin immune system, they support the notion that different natSpTrf proteins have different functions, including acting indirectly as partners in opsonization (35, 36, 38) and perhaps in immune functions other than opsonization, although the rSpTrf proteins reported here were not tested for anti-bacterial activity.

### 4.1 Protein stability, dimerization, and function correlate with glycosylation

rSpTrf-E1-Ec shows both functional similarities and differences compared to the rSpTrf proteins expressed in insect cells. rSpTrf-E1-Ec binds to *Vibrio diazotrophicus*, but does not augment phagocytosis by phagocytes above base-line (38). Similarly, rSpTrf-E1::beads bind to and are taken up by phagocytes, but do not enhance the phagocytosis of these beads. However, during shorter incubation times for phagocytes spun onto slides prior to the addition of beads, significantly more cells associate with rSpTrf-E1::beads compared to control beads. This discrepancy between shorter and longer incubation times may be due to more time for more phagocytes to interact with control beads, potentially masking early distinctions between the two types of beads. Compared to rSpTrf-E1, rSpTrf-E1-Ec is not glycosylated and is not stable, and its instability is exacerbated by freeze thaw cycles resulting in dimers or multimers (35) with a loss of function for binding targets (40). All of the rSpTrf proteins produced by insect cells are glycosylated and are stable over long periods, withstand multiple freeze thaw cycles, tend not to multimerize, and maintain function. In contrast to rSpTrf-E1-Ec, dimerized rSpTrf-E2-4 maintains function for binding and phagocytosis of beads by both polygonal and small phagocytes that is similar to the monomeric rSpTrf-E2-3. Although phagocytes bound fewer rSpTrf-E2-4::beads relative to those phagocytosed, this may be an outcome of more rapid uptake of the dimer by the phagocytes compared to the monomer of essentially the same protein. rSpTrf-E2-4::beads conferred greater phagocytosis compared to beads cross-linked with the other proteins. This suggests that the process of dimerization or multimerization of the natSpTrf proteins in sea urchin CF alters their function and redirects them from binding microbes or PAMPs to recognition, binding, and phagocytosis by polygonal and small phagocytes. This presents the possibility of dual functions of a subset of natSpTrf proteins for binding to microbes and then binding to phagocytes resulting in efficient clearance of microbes.

The rSpTrf proteins produced by insect cells and the natSpTrf proteins secreted by sea urchin phagocytes are glycosylated, although it is not known whether the N-linked oligosaccharides added by these two types of cells are similar. While other types of post-translational modifications to the proteins by both types of cells are not known, they are likely present due to the relatively large molecular weights of the recombinant proteins after the removal of N-linked oligosaccharides. Antibody blocking of rSpTrf-E2-3::beads prior to cell surface binding and phagocytosis was only effective after the proteins were deglycosylated for N-linked oligosaccharides. This suggests that the locations and/or sizes of the oligosaccharides on rSpTrf-E2-3 may interfere with binding by one or more of the rabbit-anti-natSpTrf antibodies. Furthermore, deglycosylation of both rSpTrf-E1::beads and rSpTrf-E2-3::beads results in fewer phagocytes associated with beads irrespective of whether the proteins are blocked with anti-natSpTrf antibodies. This not only suggests a basis for structural stability and function of the proteins, but infers the involvement of oligosaccharides with natSpTrf protein function and immunity in sea urchins.

### 4.2 The rSpTrf proteins have different functions

Predictions of functional differences among the SpTrf proteins have been based on their sequence diversity (35, 36) and variations in expression levels of the genes encoding proteins of specific element patterns (30). The results presented here begin to address this hypothesis. During relatively short incubations, where only cell surface binding is assessed, a significantly greater number of phagocytes are associated with all rSpTrf::beads. This suggests that the cells have a mechanism for recognizing the rSpTrf proteins on their surfaces that may not necessarily induce phagocytosis. However, when cells are incubated with rSpTrf::beads in solution over longer periods, variations in both recognition leading to binding and phagocytosis are observed among some of the rSpTrf proteins. They show several levels of function for mediating binding and phagocytosis of beads by phagocytes. For example, rSpTrf-E2-4 and rSpTrf-E2-3, that have identical sequences but differences in dimerization, show the highest level of binding and phagocytosis. rSpTrf-A6 and rSpTrf-01 that are the largest and smallest proteins, respectively, have somewhat lower levels of activity. rSpTrf-E1, -C1, and -D1 generally show base-line, constitutive binding and phagocytosis that tend to be the same as the control BSA::beads. This infers that natSpTrf in sea urchins with different element patterns may have a variety of functions, of which some may not be involved with opsonization. rSpTrf-E1-Ec binds to *Vibrio diazotrophicus* but does not activate phagocytosis, and this may also be the case for rSpTrf-E1, -C1, and -D1. In the sea urchin immune system, native SpTrf proteins with E1, C1 and D1 element patterns may act as partners with other natSpTrf proteins to augment opsonization that leads to phagocytosis by phagocytes (38). However, different possible functions for rSpTrf proteins with these element patterns have not been explored. For example, some natSpTrf proteins may interact with microbial commensals that are present on the sea urchin surface to curtail excessive proliferation of opportunistic proliferation that may become potential pathogens (51, 71). natSpTrf proteins are also expressed by cells that are located in the gut walls (49) and the proteins may be secreted into the gut lumen and function to control the intestinal microbiome (72). Interactions between the sea urchin immune system and microbial assemblages associated with these animals has been demonstrated by the ablation of specific immune components that result in changes to the associated microbiomes (73).

The phagocytes collected from different sea urchins exhibit wide ranges in their abilities to bind and phagocytose beads cross-linked with rSpTrf proteins. These differences could be due to several possibilities. Sea urchins are collected from outbred, wild populations of animals that inhabit the west coast of North America, and *S. purpuratus* shows significant genetic differences among individual animals (74). There are also differences in the *SpTrf* gene family composition as inferred from *SpTrf* gene sequences that are not shared among individual sea urchins (31). This leads to the hypothesis that cells from different sea urchins may bind optimally to the natSpTrf proteins that are secreted by that particular animal. Consequently, cells from some animals may not be able to bind well to some of the rSpTrf proteins that originated from coelomocytes collected from different sea urchins. For example, cells collected from some sea urchins show greater association with rSpTrf-E1::beads that is similar to elevated binding by the SpTrf-E2 proteins, while cells from other sea urchins show association with rSpTrf-E1::beads that is similar to BSA::beads. This is an additional level of cellular complexity in the population and the proposed functional variations of the natSpTrf proteins in individual sea urchins expands the robustness of the echinoid immune system.

### 4.3 Different types of phagocytes have different functions

The large phagocytes in *S. purpuratus* are the major types of coelomocytes in the CF, making up 40% to 80% of the cells (reviewed in (64)). These cells respond to immune challenges and have been characterized as the cells that govern cellular immune responses in the purple sea urchin (75). The polygonal and discoidal phagocytes are distinct cell types with different cytoskeletal morphologies (61). They show differences in the expression of natSpTrf proteins (37), and they do not differentiate from one to the other once they appear in the CF. Coelomocytes are terminally differentiated cells that do not proliferate in the CF once they leave their sites of hematopoiesis (76), they cannot be induced to proliferate in culture (77, 78), and they do not express the transcription factors that regulate coelomocyte proliferation (39). The small and polygonal phagocytes show increased numbers and/or augmented expression of natSpTrf in response to immune challenge (37, 39, 49, 79). The results presented here suggest new and noteworthy information regarding the three types phagocytes and their capacities to interact with the different rSpTrf proteins cross-linked to beads. i) Polygonal phagocytes have been noted with more than 70 associated beads and are the major phagocytic cell in sea urchins, in agreement with a previous report (75). They show specificity for binding and phagocytosis for a subset of rSpTrf proteins cross-linked to beads, which infers specificity for binding specific natSpTrf proteins in the CF. ii) The discoidal phagocytes are also phagocytic, but do not differentiate among the rSpTrf::beads and the BSA::beads. This may be interpreted as base-line constitutive phagocytosis of foreign particles in the absence of specific recognition of the SpTrf proteins. Discoidal cells also do not phagocytose yeast opsonized by SpC3 (65). Perhaps discoidal phagocytes have other functions in the immune system besides phagocytosis based on other opsonins bound to foreign targets. iii) The small phagocytes are phagocytic and take up rSpTrf::beads. These are the only cells to mount SpTrf proteins on the cell surface (37), which is also known for *H. erythrogramma* that has HeTrf on the surface of small phagocytes (25). The presence of natSpTrf on the cell surface and rSpTrf::beads that are phagocytosed by these cells suggests the possibility of an interaction between the rSpTrf proteins on beads and the natSpTrf proteins on the surface of small phagocytes that leads to phagocytosis. This notion is based on how readily the native Trf proteins multimerize, which is consistent for these protein in several species of sea urchins (25, 26, 37).

## 5 Conclusions

Of the four classes of sea urchin phagocytes, the polygonal and discoidal cells – or the large phagocytes – have generally been evaluated together and considered as a group as the major immune response cells in *S. purpuratus* (75). However, the activities of the large phagocytes presented here clearly differentiates the functions of these two types of cells. The polygonal phagocytes show specific binding and phagocytosis for a subset of rSpTrf::beads, whereas the discoidal cells show base-line, constitutive phagocytosis of all rSpTrf::beads. Although little is known about the small phagocytes (37, 50), they have been labeled as a member of the phagocyte class of coelomocytes strictly based on their morphology (39, 80). However, their capabilities to phagocytose rSpTrf::beads establishes them as phagocytes with inferences of immune functions, which is in agreement with their increase in cell numbers in response to immune challenges (37, 39, 79).

Phagocytosis and clearance of microbes from sea urchin hosts is a core function of the innate immune system in echinoids ((75, 81); reviewed in (82)). This process is universal and undertaken by most types of cells in animals, but is carried out most efficiently by professional phagocytes (83). A core aspect of phagocytosis is exemplified by the structure of the sea urchin complement system, which does not include a homologous terminal pathway for pathogen lysis, and therefore acts as an efficient opsonization system that results in phagocytosis (65, 84, 85). The importance of phagocytosis in immunity was first recognized by Elie Metchnikoff, which he demonstrated in sea star larvae (86), and is essential in other echinoderms for immune defense (66, 87). The process of phagocytosis and the proteins that are involved have been identified from transcriptomics of sea urchin coelomocytes (66). Opsonins that bind to non-self particles function as molecular linkages between a particle to be phagocytosed and the surface of the phagocyte that is an essential step in the process of phagocytosis. Our findings for the rSpTrf proteins cross-linked to inert beads infer that a subset of natSpTrf proteins in sea urchins have opsonin functions. Different versions of the rSpTrf::beads result in different levels of binding and phagocytosis by the different types of phagocytes, suggesting that different versions of the natSpTrf have a range of functions, of which one is opsonization to augment phagocytosis. There may be a variety of other activities carried out by the natSpTrf proteins that contribute to the effective host defense in echinoids. The complexity of the natSpTrf system in *S. purpuratus* is a combination of i) inferred variations in the functions of the natSpTrf proteins, ii) differences in phagocytosis functions among three types of phagocytes, and iii) genetic diversity among individual sea urchins within the population inhabiting the eastern Pacific ocean (74) that may be the basis for variations in phagocyte detection of SpTrf proteins. An integration of the complexities of SpTrf proteins, the phagocytes, and the genetic diversity among sea urchins results in an extraordinarily robust and flexible clearance system in sea urchin immunity for detecting and removing foreign particles including pathogens.

## Data availability statement

The original contributions presented in the study are included in the article/**Supplementary Material**. Further inquiries can be directed to the corresponding author.

## Ethics statement

The requirement of ethical approval was waived by George Washington University Institutional Review Board for the studies involving animals because Ethical approval was not required because research was conducted on an invertebrate animal. The studies were conducted in accordance with the local legislation and institutional requirements.

## Author contributions

RC: Conceptualization, Data curation, Formal analysis, Investigation, Methodology, Software, Visualization, Writing – original draft, Writing – review & editing. CGS: Investigation, Methodology, Writing – review & editing. LG: Funding acquisition, Resources, Writing – review & editing, Methodology. LCS: Funding acquisition, Investigation, Project administration, Supervision, Writing – original draft, Writing – review & editing.
