# Supplementary material for: Recombinant SpTransformer proteins are functionally diverse for binding and phagocytosis by three subtypes of sea urchin phagocytes
Source: Front Immunol. 2024 Apr 29;15:1372904. doi: 10.3389/fimmu.2024.1372904 (PMC11089230; doi:10.3389/fimmu.2024.1372904)
Supplement: Supplementary file 1 [file DataSheet_1.pdf]

Supplementary Material

Recombinant SpTransformer proteins are functionally diverse for binding and phagocytosis by three subtypes of sea urchin phagocytes

Ryley S. Crow, Chloe G. Shaw, Leon Grayfer, L Courtney Smith

Contents **Page**

Supplementary Figures
Figure S1 | General SpTrf protein structure. ……………………………………………………...…2
Figure S2 | The alignment of rSpTrf proteins expressed in insect cells shows the ……….…..…3
sequence diversity among proteins with different element patterns.
Figure S3 | rSpTrf-E2-4 increases in molecular weight over time. …………………………...…5
Figure S4 | The rSpTrf proteins do not multimerize with each other. ………………………..…5
Figure S5 | Incubation of live cells with antibodies at 0ºC limits antibody ………….………....6
binding to the surface by blocking penetration past the plasma membrane.
Figure S6 | Phagocytes spun onto glass slides before incubation with beads bind but ………..…7
do not phagocytose beads.
Figure S7 | Phagocytes incubated with beads in solution phagocytose beads. ……………….……8

Supplementary Tables
Table S1 | Conserved N-linked oligosaccharide positions in the rSpTrf proteins ………………9
Table S2 | Primers for Gibson Assembly of the *SpTrf* cDNA sequences and …………………10
the *pMIB* expression vector.
Table S3 | Primers for standard ligation of *SpTrf* inserts into the *pMIB* expression vector ……10

Supplementary Text Files
Text file S1 | rSpTrf-E2.1 is not expressed by Sf9 insect cells. ……………………..………11
Figures 1.1-1.6 ……………………………………………………..13 - 18
Table 1.1 ……………………………………………………………..19
Text file S2 | The initial approach to evaluate phagocytosis of rSpTrf::beads by sea …………20
urchin phagocytes used magnetic attraction of cells associated with beads.
Figures 2.1-2.3 ……………………………………...………..22 – 24

References …………………………………………………………………..…………..……25

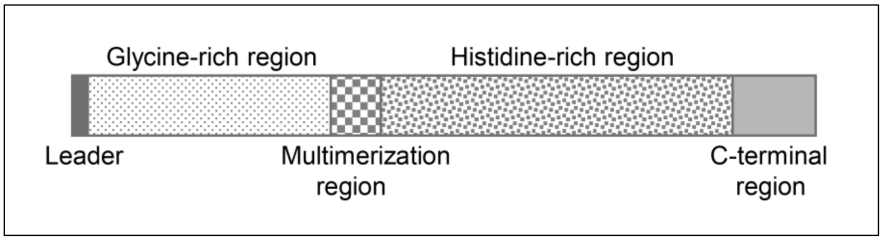

Figure S1 | General SpTrf protein structure. This figure is modified from Figure 1A in Lun et al. [1].

Figure S2 | The alignment of rSpTrf proteins shows the sequence diversity among proteins with different element patterns[1]

```
           Leader                              / Beginning of the glycine-rich region
A6   MEVKVTLIVA IVAALAIS AHA/QSDFNERR GKENGRERGQ DRFGGRPDGM QMGGPRQDGG
D1   M~VKVTLIVA IVAALAIS AHA/QRDYNELR GNKNGRERGQ GRFGGRPGGM QMGGSRQDGG
C1   MEVKVTLIVA IVAALAIS AHT/QRDYNERR GNENGRERGQ GRFGGRPGGM QMGGPRQDGG
E2   MEVKVTLIVA IVAALAIS AHA/QRDFNERR GKENDTERGQ GGFGGRPGGM QMGGPRQDGG
E1   MEVKVTLIVA IVAALAIS AHA/QRDFNERR GKENDTERGQ GGFGGRPGGM QMGGRRQDGG
01   M~VKVTLIVA IVAALAIS AHA/RRDFNERR GKENGRERGQ GGFGGRPGGM QTGSPRQDGG
                         AHA QRDFNERR GKENDTER
                  peptide used to generate the rabbit anti-natSpTrf-66 antibody

A6   PMGGRRFDGP RFGAPQMGGP RQNGGPMGGR RFDGPGFGAP PMGGPRQDGG PMGGRRFDGP
D1   PMGGRRFDGP DSGAP~~~~~ ~~~~~~~~~~ ~~~~~~~~~~ QMDGRRQDGG PMGGRRFDGP
C1   PMGGRRFDGH GFGAP~~~~~ ~~~~~~~~~~ ~~~~~~~~~~ PMGGPRQDGG PMGGRRFDGP
E2   PMGGRRFDGP ESGAP~~~~~ ~~~~~~~~~~ ~~~~~~~~~~ ~~~~~~~~~~ ~~~~~~~~~~
E1   PMGEMRFDGP ESGAP~~~~~ ~~~~~~~~~~ ~~~~~~~~~~ ~~~~~~~~~~ ~~~~~~~~~~
01   PMGGMRFDGP ESGAP~~~~~ ~~~~~~~~~~ ~~~~~~~~~~ ~~~~~~~~~~ ~~~~~~~~~~

                                   End of the glycine-rich region  /  Multimerization region
A6   GFGAPQMGGP RQNGGPMGGR RFDGPGFGGS RPDGAGGRPF F/GEGGRRGDG EEETDAARQI
D1   GFGAPEMDGR RQNGGPMGGR RFDGPGFGGS RPDGAGGRPF F/GQGGRRGDG EEETDAAQQI
C1   GFGTPQMDGR RQNGGPMGGR RFDGPRFGGS RPDGAGGRPF F/GQGGRRGDG EEETDAAQQI
E2   ~~~~~QMEGR RQNGGPMGGR RFDGPRFGGS RPDGAGGRPF F/GQGGRRGDG EEETDAAQQI
E1   ~~~~~QMDGR RQNGGPMGGR RFDGPVFGGS RPDGAGGRPF F/GQGGRRGDG EEETDAAQQI
01   ~~~~~QMDGR RQNGGPMGGR RFDGPRFGGS RPDGTGGRPF F/GQGGRRGDG EEETDAAQQI
                                                    GGRRGDG EEETDAAQQI
                                     peptide used to generate the rabbit anti-natSpTrf-68 antibody

        Multimerization region     / Beginning of the histidine-rich region
A6   G~~~~~PGRF DGPGHGHY/GH HQGAGRPFFG NPPPFN~~~~ ~~~~~~~~~~ ~~~~~~~~~~
D1   GDGPGGPGQF DGPGRRHH/GH RQG~~~~~~~ ~~~~~~~~~~ ~~~~~~~~~~ ~~~~~~~~~~
C1   GDGLGGSDRF DGPRRGHH/GH RQG~~~~~~~ ~~~~~~~~~~ ~~~~~~~~~~ ~~~~~~~~~~
E2   GDGLGGRGQF DGHGRGHH/GH RQG~~~~~~~ ~~~~~~~~~~ ~~~~~~~~~~ ~~~~~~~~~~
E1   GDGLGGRGQF DGPGRRHH/GR KPFGDRPFGR ~~~~RNHT EGHQGHNETG NETGDHPHDGHG
01   GDGLGGPGQF DGPGRRHH/GR KPFGDRPFGR ~~~~RNHT EGHQGHNETG NETGDHPH~~~~
     GDGL (continued peptide for α-natSpTrf-68)                 unlikely

A6   ~~~~~~~~ ~~~~~~~~~~PEQ EP~~RNDSSE EDGRHHRHHD RHHAHHGHHG HHEHHHQHHN
D1   ~~~~~~~~ HPQDQ~~~~AEEQ PFGQRNESSE EDGRPHPHHH RHH~~~GHHH RHH~~~~~~N
C1   ~~~~~~~~ PPQDR~~~~PEEQ PFGQRNYSSE EDGRPHPHHH RHH~~~GHHR HHHHH~~~~N
E2   ~~~~~~~~ PPQDR~~~~PEEQ PFGQRNESSD EDGRPHPRHH ~~~~~~GRHH QHHHR~~~~N
E1   RGHHGHRQ          PEEQ PFGQRNESSD EDGRPHPRHH ~~~~~~GRHH QHHHR~~~~N
01   ~~~~~~ ~~~~~~~~~~~~~~~ ~~~~~~~~~~ ~~~~~~~~~~ ~~~~~~~~~~ ~~~~~~~~~~
                                                                    unlikely
```

```
A6  HTEGHQ~~~~ ~~~~~~~~~~ ~~~~~DHDRP MFEMRPFRFN PLGRKPFGDH PFGRRNHTEG
D1  HTEGHQGHNE TG~~~~~~~ ~~~~DQDQDK~ LHDTRPFRYN HFGRKPFGDR PFGRRNHTEG
C1  QTEGHQGHNE TG~~~~~~~ ~~~~DQDQDKP I~DTRPFRFN HFGRKPFGGR PFGRRNHTEG
E2  HTEGHQGHNE TGDHPHRHHN KTGDGDQDRP MFEMRPFRFN PFGRKPFGDR PFGRR~~~~~
E1  HTEGHQGHNE TGDHPHRHHN KTGDGDQDRP MFEMRPFRFN PFGRKPFGDR PFGRR~~~~~
01  ~~~~~~~~~~ ~~~~~~RHHN KTRDGDQDRP MFEMRPFRFN PFGRKPFGDR PFGRR~~~~~

A6  HQGHNETGDH PHRHHSKNVD GDQDTGHHGH HGHHEHHHHQ HDHREGHQDH DRPMFEMRPF
D1  HRGHNETGDH PHRHHNKTRD GDQD~~~~~~ ~~~~~~~~~~ ~~~~~~~~~~ ~RPMFEMRPF
C1  HQGHNETGDH PHRHHNKTGD GDQD~~~~~~ ~~~~~~~~~~ ~~~~~~~~~~ ~RPMFESRPF
E2  ~~~~~~~~~~ ~~~~~~~~~~ ~~~~~~~~~~ ~~~~~~~~~~ ~~~~~~~~~~ ~~~~~~~~~~
E1  ~~~~~~~~~~ ~~~~~~~~~~ ~~~~~~~~~~ ~~~~~~~~~~ ~~~~~~~~~~ ~~~~~~~~~~
01  ~~~~~~~~~~ ~~~~~~~~~~ ~~~~~~~~~~ ~~~~~~~~~~ ~~~~~~~~~~ ~~~~~~~~~~

A6  RFNPLGRKPF GDHPFGRRNH TEGHQGHNET GDHPHRHHSK TGDGDQDRPM FETRPFWVNP
D1  RFNPFGRKPF GGRPFDRR~~ ~~~~~~~~~~ ~~~~~~~~~~ ~~~~~~~~~~ ~~~~~~~~~~
C1  RFNPFGRKPF GDRLFGRR~~ ~~~~~~~~~~ ~~~~~~~~~~ ~~~~~~~~~~ ~~~~~~~~~~
E2  ~~~~~~~~~~ ~~~~~~~~~~ ~~~~~~~~~~ ~~~~~~~~~~ ~~~~~~~~~~ ~~~~~~~~~~
E1  ~~~~~~~~~~ ~~~~~~~~~~ ~~~~~~~~~~ ~~~~~~~~~~ ~~~~~~~~~~ ~~~~~~~~~~
01  ~~~~~~~~~~ ~~~~~~~~~~ ~~~~~~~~~~ ~~~~~~~~~~ ~~~~~~~~~~ ~~~~~~~~~~

                    / C-terminal region
A6  FGRRPFGDRP FDRR/NGTEEG SPRRDGHPHP HGNRGRWGEN ESEEKEHPTT ESVTTFSPLK
D1  ~~~~~~~~~~ ~~~~/NGTEEG SPRRDGHRRP YGNRGRWGEN ESEEKEHPTT ESVTTSSPPE
C1  ~~~~~~~~~~ ~~~~/NGTEEG SPRRDGHRRP YGNRGRWGEN ESEEKEHPTT ESVTTSSPPE
E2  ~~~~~~~~~~ ~~~~/NGTEEG SPRRDGQRRP YGNRGRWGEN ESEEKEHPTM ESVTTSSPP~
E1  ~~~~~~~~~~ ~~~~/NGTEEG SARRDGQRRP YGNRGRWGEN ESEEEEHPTT ESVTTSSPP~
01  ~~~~~~~~~~ ~~~~/NGTEEG SPRRDGQRRP HGNRGRWGEN ESEEKEHPTT ESVTTSSPPE
                     GTEEG SPRRDGQRRP YGNR    unlikely
        peptide used to generate the rabbit anti-natSpTrf-71 antibody

A6  VIEIAINEVD TNVVAEV
D1  VV~~AINEED INVVAEV
C1  VVEIAFNEED VNVVAEV
E2  .......... .......
E1  .......... .......
01  VVEIAIND~~ ~~~VAEV
```

[1]The alignment shows the similarities and differences among the SpTrf protein sequences chosen for expression in insect cells. Proteins are defined based on their element pattern. The alignment also shows the conserved sites for **N**-linked oligosaccharides including those that are unlikely to be linked to oligosaccharides (see Table S3). Peptides used to produce the rabbit-anti-natSpTrf antibodies are indicated on the bottom lines of the alignment [2]. The **G** at location 276 in some *SpTrf-E2* messages are edited in sea urchin cells from encoding a glycine to a stop codon resulting in truncated SpTrf-E2.1 proteins [3, 4].

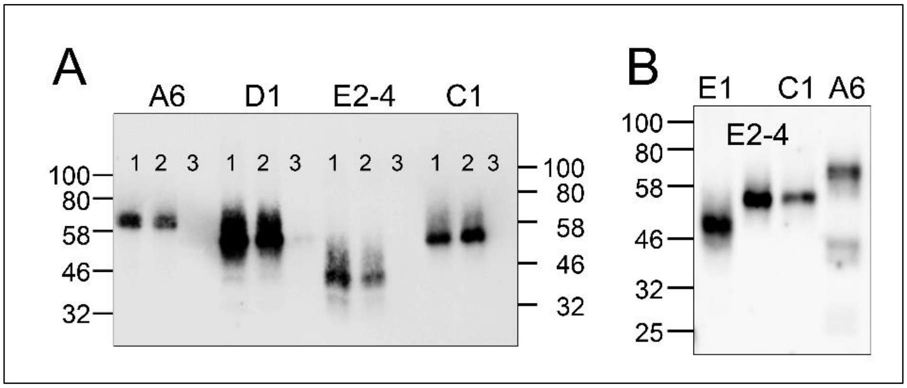

Figure S3 | rSpTrf-E2-4 increases in molecular weight over time. **(A)** A subset of rSpTrf proteins from a preliminary protein isolations prior to scaling up the culture volumes are evaluated for expression by Western blot. rSpTrf-E2-4 is ~40 kD and smaller than the other proteins on the blot. There are three lanes for each rSpTrf protein that show subsequent elutions from the Ni-NTA agarose beads (lanes 1, 2), and a sample from the media after protein isolation by nickel affinity (lane 3). **(B)** After the large scale isolation of rSpTrf proteins and storage at -80°C for about five months, rSpTrf-E2-4 shows a molecular weight increase to about 55 kDa and is the same size as rSpTrf-C1. Both Western blots were evaluated with rabbit-anti-V5-HRP (3000X dilution: Invitrogen) followed by ECL incubation and imaging in a GelDoc Touch (BioRad) imager.

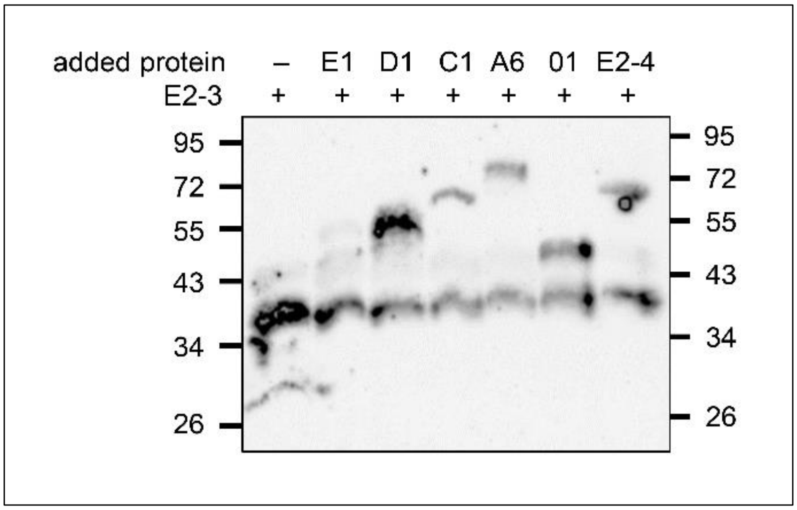

Figure S4 | The rSpTrf proteins do not multimerize with each other. rSpTrf-E2-3 was incubated with each of the other rSpTrf proteins (protein, which) at rt for 1 hr. Equal mass quantities of 1.5 µg for each protein was optimized for detection by Western blot. None of the proteins show an increase in size suggesting that multimerization does not occur. The blot was evaluated using rabbit-anti-V5-HRP (3000X dilution; Invitrogen) and imaged as in Figure S3.

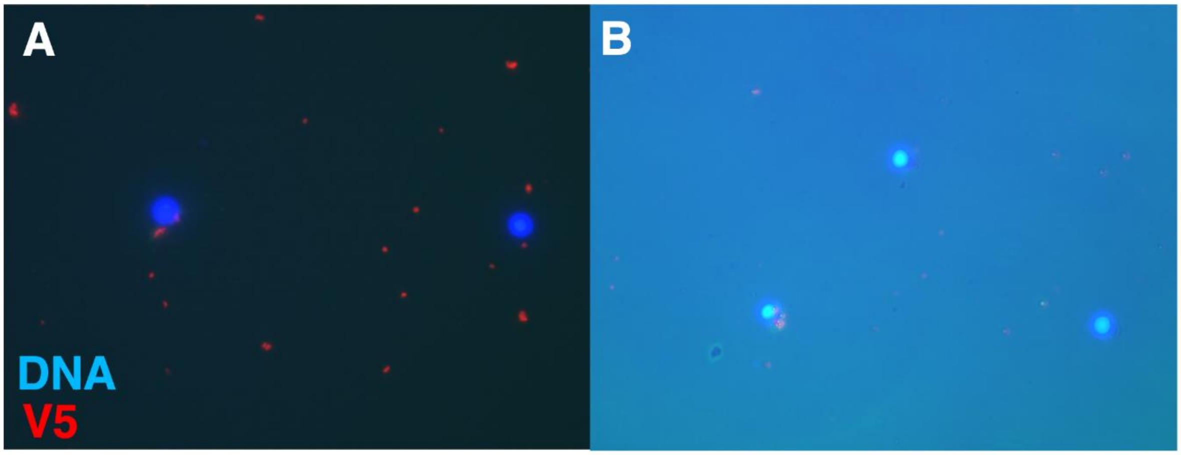

Figure S5 | Incubation of live cells with antibodies at 0ºC limits antibody binding to the surface by blocking penetration past the plasma membrane. Mouse-anti-actin antibody does not bind to the actin cytoskeleton when incubated with live coelomocytes 0°C before fixation and permeabilization. The antibody is blocked from passing through the plasma membrane. The two panels show coelomocytes from two different sea urchins **(A**, **B)**. DAPI (blue) labeling indicates the nucleus of the cells. The rSpTrf::beads (red), bound by the rabbi-anti-V5-549 (3000X dilution; Invitrogen), are on the surface of the cells. The brightfield merge for the cells from sea urchin **(A)** is unavailable. The brightfield image for the cells from animal **(B)** is shown to visualize the beads. All beads appear red indicating that are all bound to the surface of the cells. Lack of green labeling indicates that the mouse-anti-actin (1500X dilution; Invitrogen) is blocked from binding cytoskeletal actin in non-permeabilized cells and thus the goat-anti-mouse-Ig-488 (6000X; Invitrogen) antibody is negative after cells are fixed and permeabilized.

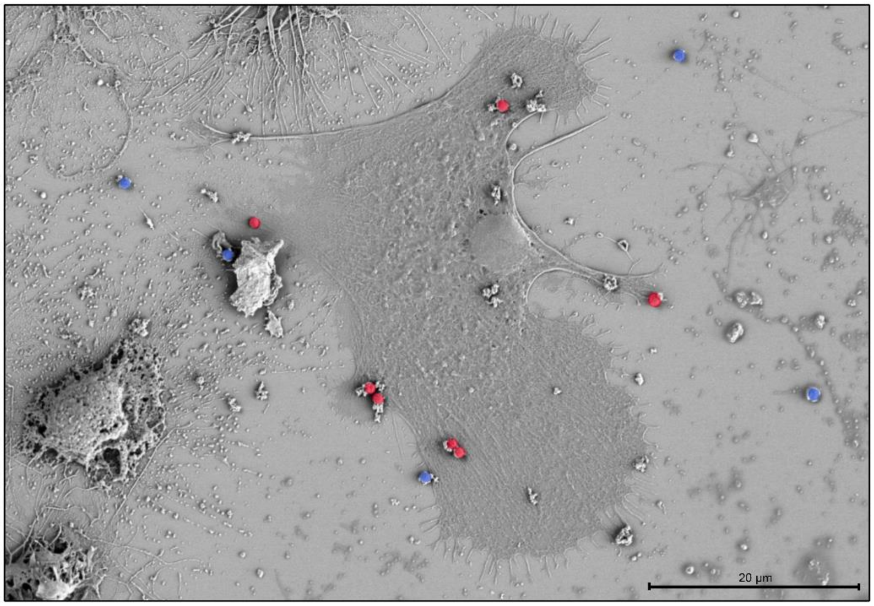

Figure S6 | Phagocytes spun onto glass slides before incubation with beads bind but do not phagocytose beads. Centrifuged cells are extremely flattened that limits cytosolic space for phagocytosis. The polygonal cell shown by scanning electron microscopy occupies 22% of the field of view area. Notably, more beads (false red color , n = 7) are observed on the cell surface than on the glass slide (false blue color, n = 5) in 78% of the area suggesting an interaction between the cell surface and the rSpTrf-E2-3::beads.

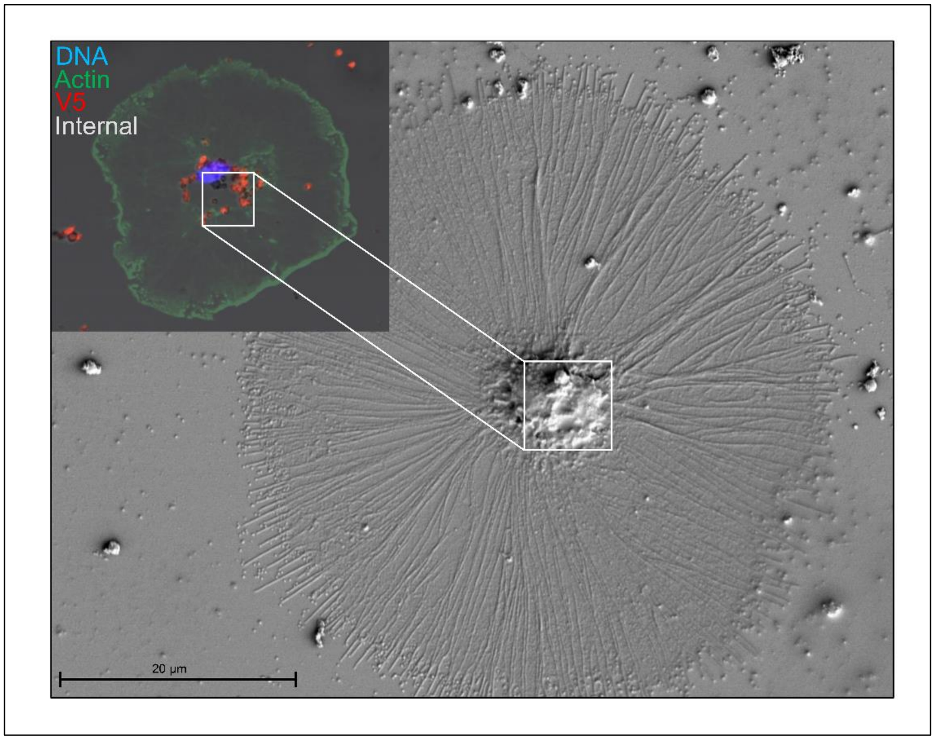

Figure S7 | Phagocytes incubated with beads in solution phagocytose beads. The scanning electron microscopy image shows a discoidal cell incubated with rSpTrf-E2-3::beads in solution. It shows a large irregular, lumpy region around the nucleus suggesting internalized beads. For comparison, the insert shows a discoidal cell by fluorescence microscopy with both surface bound and phagocytosed beads. Red beads are on the cell surface and black unlabeled beads are internal. Internal beads are positioned around the nucleus in a similar location as the irregular region at the center of the cell imaged by SEM.

Supplementary Tables

Table S1 | Primers for Gibson Assembly of the *SpTrf* cDNA sequences and the *pMIB* expression vector

Primer[1]	Sequence[2]	Length	Tm[3]	Target
01F-pMIB	GTATACATTTCTTACATCTATGCC<br>CAAAGCGATTTCAATGAACGAC	46	65.4	*01*
01R-pMIB	GGAGAGGGTTAGGGATAG<br>CACCTCGGCGACATCATT	36	69	
pMIBF-01	CAATGATGTCGCCGAGGTG<br>GGTAAGCCTATCCCTAACC	38	69.9	*pMIB* for *01*
pMIBR-01	GTCGTTCATTGAAATCTCTTCG<br>GGCATAGATGTAAGAAATGTATAC	46	65.4	
E1F-pMIB[2]	GTATACATTTCTTACATCTATGCC<br>CAAAGAGATTTCAATGAACGACG	47	65.4	*E1*, *E2*, *E2.1*
E1R1-pMIB	GGAGAGGGTTAGGGATAG<br>AGGTGGTGAAGATGTCGTTAC	39	68.7	*E1*
E2R-pMIB	GGGTTAGGGAGAGGCTTACC<br>AGGTGGTGAAGATGTCGTTAC	41	70.5	*E2*
E2.1R-pMIB	GGGTTAGGGAGAGGCTTACC<br>ATGACCATCAAACTGACCGCG	41	71.5	*E2.1*
pMIBF-E1	GTAACGACATCTTCACCACCT<br>GGTAAGCCTATCCCTAACCC	41	69.5	*pMIB* for *E1*
pMIBR-E1	CGTCGTTCATTGAAATCTCTTTG<br>GGCATAGATGTAAGAAATGTATAC	47	66.3	*pMIB* for *E1*, *E2*, *E2.1*
pMIBF-E2	GTAACGACATCTTCACCACCT<br>GGTAAGCCTATCCCTAACCCT	42	71.1	*pMIB* for *E2*
AC0F-pMIB	GTATACATTTCTTACATCTATGCC<br>CAAAGCGATTTCAATGAACGAC	46	65.4	*A6*, *C1*, *01*
ACR2-pMIB	GGTTAGGGATAGGCTTACC<br>CACCTCGGCGACCACAT	36	70.1	*A6*, *C1*
pMIBF-A	CAATGTGGTCGCCGAGG<br>GGTAAGCCTATCCCTAAC	34	68	*pMIB* for *A6*
pMIBR-A	GTCGTTCATTGAAATCGCTTTG<br>GGCATAGATGTAAGAAATGTATAC	46	65.4	
D1F-pMIB	GTATACATTTCTTACATCTATGCC<br>CAAAGAGATTACAATGAACTACG	47	64.5	*D1*
D1R-pMIB	GGAGAGGGTTAGGGATAG<br>CACCTCAGCGACCACATT	36	69	
pMIBF-D1	AATGTGGTCGCTGAGGTG<br>GGTAATCCTATCCCTAACCC	38	68.8	*pMIB* for *D1*
pMIBR-D1	ACGTAGTTCATTGTAATCTCTTTG<br>GGCATAGATGTAAGAAATGTATAC	48	64.6	
C1F-pMIB	GTATACATTTCTTACATCTATGCC<br>CAAAGAGATTACAATGAACGACG	47	65.4	*C1*
C1R-pMIB	GGAGAGGGTTAGGGATAG<br>CACCTCGGCGACCACAT	35	70.3	
pMIBF-C1	AATGTGGTCGCCGAGGTG<br>GGTAAGCCTATCCCTAACC	37	70	*pMIB* for *C1*
pMIBR-C1	CGTCGTTCATTGTAATCTCTTTG<br>GGCATAGATGTAAGAAATGTATAC	47	65.4	

[1]The primer name indicates the amplicon, and the target into which it will be ligated by Gibson Assembly.

[2]The upper and lower sequences correspond to the two DNA fragments that are ligated by Gibson Assembly.

[3]Tm calculations are based on www.biopho.org/minitools/melting temperature/demo

Table S2 | Primers for standard ligation of *SpTrf* inserts into the *pMIB* expression vector

Primer	Sequence	Length	Tm	Target
E2F1	CCGaagcttACAAAGAGATTTCAATGAA[1]	28	55.5	*E2, E2.1*
E2R1	GCCctcgagAGGTGGTGAAGATGTCGT[2]	27	64.3	*E2*
E2.1R1	GCGctcgagATGACCATCAAACTGACC[2]	27	62.8	*E2.1*

[1]Lower case nucleotides indicate the *Hind*III restriction site.
[2]Lower case nucleotides indicate the *Xho*I restriction site.

Table S3 | Conserved N-linked oligosaccharide positions in the rSpTrf proteins

	Number of positions	
**Protein**	**N**[1]	**NetNGlyc**[2]
A6	10	7
D1	8	7
C1	8	7
E2	7	5
E1	8	7
01	5	4

[1]Conserved sites for the addition of N-linked oligosaccharides are N-X(not P)-T/S.
[2]NetNGlyc is an on-line tool (https://services.healthtech.dtu.dk/services/NetNGlyc-1.0/) used to estimate the potential for glycosylation at the N positions. See the rSpTrf alignment (Figure S2) for the locations of asparagines (**N**) that could be modified with N-linked oligosaccharides.

Supplementary Text File S1

rSpTrf-E2.1 is not produced by Sf9 insect cells

The SpTrf-E2.1 version of the SpTrf-E2 protein is a truncated protein that is expressed and secreted by coelomocytes of the sea urchin, *Strongylocentrotus purpuratus* [3, 5]. The mRNA from the *SpTrf-E2* gene has a GGA encoding a glycine that is edited to, which is a stop translation codon [3]. This stop, that is present in the sequences of many cDNAs generated from mRNAs isolated from individual animals, is not present in the corresponding genes, suggesting editing of these specific messages [4]. The resulting deduced truncated protein is 14.6 kDa, and is missing most of the C terminal region that is the majority of the histidine-rich region (see above, Supplementary Figure S1). Because edited mRNAs encoding truncated SpTrf proteins decrease in prevalence upon immune challenge, it has been postulated that they may serve a surveillance function that is different from the full length proteins, of which some bind to pathogens ([6]; reviewed in [7]). To test this hypothesis, rSpTrf-E2.1 was included with the other rSpTrf proteins for production in insect cells for the purpose of characterizing protein function.

The insert from the cDNA clone 2-2439 (GenBank accession number EF065832) [3]) encodes a SpTrf protein with an E2 element pattern and was amplified by PCR to generate either the full length SpTrf-E2 protein or the truncated SpTrf-E2.1 protein using different reverse primers (Table 1.1). The amplicons were ligated into the *pMIB*/*V5-HisA* expression vector (ThermoFisher) using the Gibson Assembly method (New England Biolabs), transfected, grown, and isolated from *E. coli*, followed by lipofection into Sf9 insect cells with Cellfectin II reagent (Invitrogen). The rSpTrf-E2.1 and rSpTrf-E2-3 proteins were isolated from the cell culture media by nickel affinity as described in the main paper [8, 9]. When expression of rSpTrf-E2.1 by insect cell cultures was evaluated by Western blot, three different cultures failed to yield rSpTrf-E2.1 protein although rSpTrf-E2-3 was expressed and isolated successfully (Fig. 1.1). The same result was obtained when using different primary antibodies to evaluate the Western blot; either rabbit-anti-V5-HRP or a mixture of three rabbit-anti-natSpTrf antibodies.

To determine the basis for the expression failure of rSpTrf-E2.1, transfected Sf9 cells were first evaluated for whether the expression vector was incorporated into the genome. Genomic DNA (gDNA) was isolated from three cultures of insect cells; Sf9 cells transfected with *pMIB*/*V5-HisA* expression vector ligated with the E2.1 sequence (*pMIB-E2.1*), or transfected with *pMIB-E2-3* as the positive control, and Sf9 cells that were not transfected with an expression vector and served as the negative control. gDNA from all samples appeared to be of high quality, did not show protein contamination (Fig. 1.2A-C), were composed of large strands of DNA of greater than 10 kb (Fig. 1.2D), and supported PCR amplification of the cytoplasmic actin gene (Fig. 1.3A; Table 1.1). Amplification of the *pMIB* constructs from the gDNA template using either *pMIB* primers (Table 1.1) or *SpTrf* primers (Fig. 1.3D) indicated that both expression constructs, *pMIB-E2.1* and *pMIB-E2-3*, were incorporated into the gDNA of the Sf9 insect cells (Fig. 1.3C). Furthermore, there was no false positive amplification from the Sf9 control cells that had not been transfected with a construct (Fig. 1.3B). Amplicons produced from the gDNA were the same size as those produced from the expression vectors (Fig. 1.3C). These results indicated that the *pMIB* expression constructs were transfected into the Sf9 insect cells and that they integrated into the genome.

Given that the Sf9 cells contained the transgenes, expression of the *pMIB* constructs were evaluated to determine whether *pMIB-E2.1* was expressed compared to *pMIB-E2-3*. Total RNA

was isolated from $5X10^6$ cells from each culture using Trizol (Invitrogen). Spectrophotometry and gel analysis indicated that total RNA was isolated successfully (Fig. 1.4A-C) and that rRNA bands were observed on a gel (Fig. 1.4D). The feint smear in the gel lanes suggested the presence of mRNA. Total RNA (0.36 – 0.98 µg) was reverse transcribed with qScript cDNA synthesis kit (Quanta Bio) and evaluated by PCR for the presence of *rSpTrf-E2.1* and *rSpTrf-E2-3* messages. Results indicated that Sf9 cells transfected with either *pMIB-E2.1* or *pMIB-E2* both had RNA corresponding to the vector inserts (Fig. 1.5). This was evaluated using the SpTrf primers (Fig. 1.3D) and the primers used for Gibson Assembly (Table 1.1) and results were consistent with transgene expression. Furthermore, the amplicons from the RT-PCR reactions were the same size as those amplified directly from the expression constructs (Fig. 1.5B, C).

Given that the *pMIB* expression vectors were transfected and incorporated into the Sf9 genome, and that they drove expression of the transgenes, the last question was to determine whether the rSpTrf-E2.1 protein was produced by the Sf9 cells. Although the difference in protein isolation indicated the failure of the rSpTrf-E2.1 protein to be secreted, it did not show whether the protein was produced and secreted and then degraded in the culture media prior to isolation, whether it was produced and degraded by the cells prior to secretion, or whether it was not produced by the cells. The insect cells from the three cultures were evaluated by microscopy and the transport vesicles in the cells were quantified. To improve the visualization of vesicles among the cultures, the cells were incubated with Brefeldin A (5 µg/ml; BioLegend) for 18 hours at 27°C according to the manufacturer (see also [10]), washed in PBS to remove any secreted rSpTrf proteins, and $6X10^4$ cells were spun onto slides, fixed, and incubated with antibodies according to [2]. The primary antibodies were either i) rabbit-anti-V5-549 (3000X dilution, Rockland) or ii) rabbit-anti-natSpTrf (300X dilution, [2, 11] followed by the secondary antibody, goat-anti-rabbit Ig-555 (6000X dilution, Invitrogen). Cells were counter labeled with mouse anti-actin (1500X dilution, Invitrogen) followed by the secondary antibody goat anti-mouse Ig-488 (6000X dilution, Invitrogen), and DAPI. Background was evaluated by incubating the cells the normal rabbit serum (NRS) rather than the primary antibody. Cells were evaluated on a Axioscope fluorescence microscope (Zeiss). The percentage of cells with $V5^+$ or $SpTrf^+$ vesicles were evaluated in addition to the number of $V5^+$ or SpTrf+ vesicles per cell. Results for anti-V5 and anti-natSpTrf were the same and showed that the percentage of cells with the *pMIB-E2.1* transgene was not different from the control cells incubated with NRS or the cells that had not been transfected (Fig. 1.6A). In comparison, there were significantly more cells with $V5^+$ or $SpTrf^+$ vesicles that were transfected with the *pMIB-E2-3* expression construct. When vesicles per cell were evaluated, particularly for $\geq$ 4 vesicles per cell, the numbers of $V5^+$ or $SpTrf^+$ vesicles in cells transfected with *pMIB-E2.1* were not different from the control cells or the cells that had not been transfected (Fig. 1.6B). The control cells transfected with *pMIB-E2-3* showed significantly more $V5^+$ vesicles compared to the non-transfected control cells (Fig. 1.6C-H). This analysis suggested that the truncated rSpTrf-E2.1 protein may have been unstable and degraded by the insect cells prior to loading into transport vesicles. It is noteworthy that sea urchin coelomocytes produce and secrete this truncated version [5] indicating significant differences between insect cells and echinoid cells that may be based on post translational modifications that are addressed in the main paper.

Figures for Supplementary Text File S1

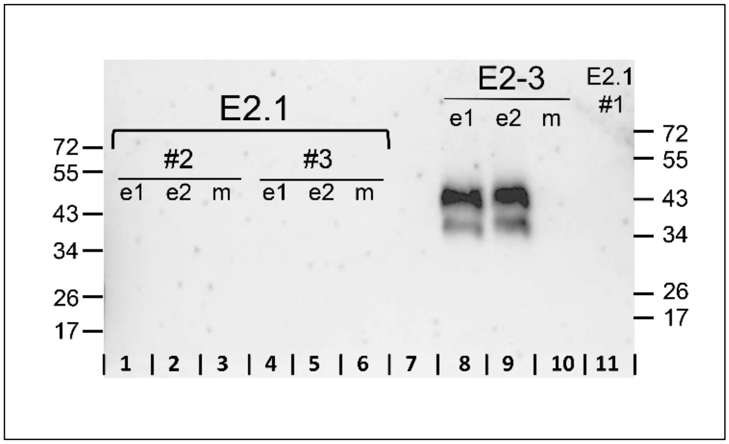

Fig. 1.1 | rSpTrf-E2.1 protein production fails repeatedly for Sf9 cell cultures. Samples from three cultures of insect cells transfected with the *pMIB-E2.1* expression construct and one culture transfected with *pMIB-E2-3* are evaluated for protein production. Results from the Western blot shows that rSpTrf-E2.1 is not isolated from culture #1 (lane 11) or from cultures #2 and #3 (lanes 1-6). Lanes 1-2 and 4-5 show the first and second elutions (e1, e2) from the Ni-NTA (Invitrogen) affinity column, and lanes 3 and 6 show the media (m) after Ni-NTA affinity protein isolation. For comparison, one insect cell culture produces rSpTrf-E2-3 (lanes 8-10). Lanes 8 and 9 are loaded with samples from the first and second elutions (e1 and e2) of rSpTrf-E2-3 from the nickel affinity column. Lane 10 shows the media (m) that after isolation by Ni-NTA affinity, in which no rSpTrf-E2-3 remains. Proteins are separated on a 15% SDS-PAGE and transferred to PVDF filters (Immobillon-P^{sq}, Millipore) by electroblotting (Trans Blot Turbo transfer system, BioRad), and the filter is evaluated with rabbit-anti-V5-HRP (3000X dilution; Invitrogen), incubated in SuperSignal^{TM} West Pico PLUS Chemiluminescent Substrate according to the manufacturer (ThermoScientific), and imaged in a ChemiDoc Touch imaging system (BioRad). When duplicate blots are evaluated with rabbit-anti-natSpTrf antibodies [11, 12], similar negative results for rSpTrf-E2.1 are obtained (not shown). Protein standard (Broad Range, ThermoFisher) sizes are indicated.

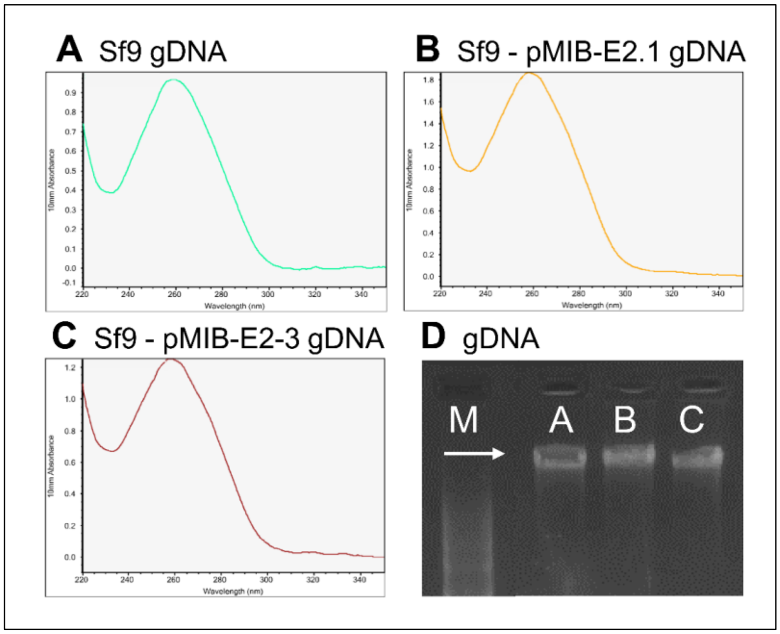

Fig. 1.2 | Genomic DNA is isolated from Sf9 cell cultures. Genomic DNA was isolated from $5X10^6$ insect cells with the GeneJET gDNA purification kit (Fermentas). **(A – C)** OD^{260} readouts by spectrophotometry (NanoDrop 2000c, ThermoScientific) are shown for the genomic DNA isolated from the three cell cultures. Scans indicate no contamination with proteins. **D**. Isolation from the Sf9 cultures yields large, non-degraded genomic DNA of >10 kb (arrow). Lane letters correspond to the scans in panels **A**, **B**, and **C**. Each lane is loaded with about 100 ng of gDNA and separated on a 0.75% agarose gel in Tris-acetate-EDTA buffer (TAE; 40 mM Tris; 20 mM acetic acid, 1 mM EDTA) with ethidium bromide. The DNA standard is loaded in lane M. The gel was imaged with the Kodak Molecular Imaging System (Kodak Gel Logic 1500) under ultraviolet light.

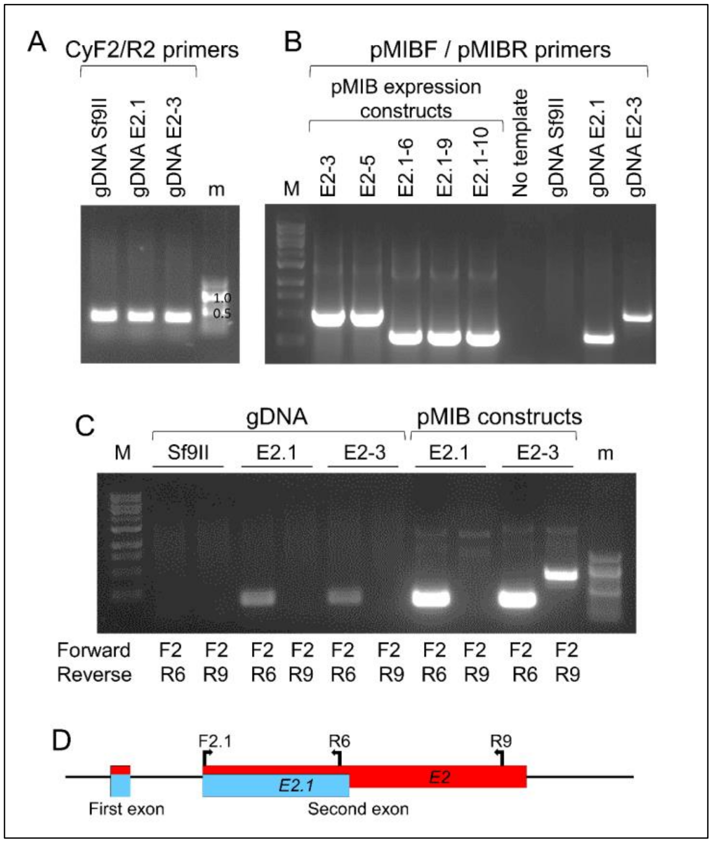

Fig. 1.3 | PCR amplification of gDNA from Sf9 insect cells indicates that the pMIB expression vectors are in-corporated into the genome. **(A)** To ensure that the Sf9 insect cell gDNA can support PCR, primers (Table S1) for cytoplasmic actin (Cy) are used to amplify the actin gene. Amplicons of the expected size are obtained. **(B)** Primers for *pMIB* show amplicons for *pMIB-E2.1*. Several *pMIB* expression constructs are evaluated for insert sizes using primers specific for *pMIB* sequences that flank the inserts. These *pMIB* constructs have the same insert sequence, but are transfected into Sf9 cells at different times followed by different isolations of the rSpTrf-E2 proteins. Both *pMIB-E2-3* and *-E2-5* constructs amplified inserts of the expected size. Three *pMIB-E2.1* constructs (*-6*, *-9*, *-10*) amplified smaller inserts of the expected size. (*pMIB-E2.1-10* was chosen for all subsequent analyses.) When the *pMIB* primers are used with the gDNA template isolated from cells transfected with *pMIB-E2-3* or with *pMIB-E2.1*, the amplicon sizes match those of the constructs. No amplicons are observed when the template is gDNA from Sf9 cells that had not been transfected with a *pMIB* expression construct or when the template is omitted. **(C)** Primers specific for *SpTrf* sequences amplify *SpTrf-E2.1* and *SpTrf-E2-3* sequences from Sf9 gDNA. To verify the results using the *pMIB* primers, *pMIB* constructs and gDNA are used as templates for primers specific for the *SpTrf* sequences. Both *F2.1*/*R6* and *F2.1*/*F9* primer pairs (Table S1) that are specific for *SpTrf* sequences, amplify the insert of the *pMIB-E2-3* construct. Because the insert for *E2.1* is truncated, only the *F2.1*/*R6* primer pair amplifies the insert. When these SpTrf primer pairs are used with gDNA from Sf9 cells transfected with *pMIB-E2.1* as the template, the amplicon generated by the *F2.1*/*R6* primers is the expected size of the *E2.1* construct insert. gDNA from Sf9 cells transfected with *pMIB-E2-3* amplified with *F2.1*/*R6* and *F2.1*/*R9* primer pairs results in amplicons of the expected sizes. gDNA from non-transfected Sf9 cells do not support amplification with *SpTrf* primers. Sizes of the DNA standards are shown in **(A)**. The gels are 0.8% agarose in TAE running buffer with ethidium bromide. The gels were imaged under ultraviolet light with the Kodak Molecular Imaging system (Kodak Gel Logic 1500). **(D)** *SpTrf* primer locations are shown. A standard structure of an *SpTrf* gene is shown. *SpTrf-E2* is indicated in red, while the truncated version, *SpTrf-E2.1*, is indicated in blue. The overlapping regions have identical sequence.

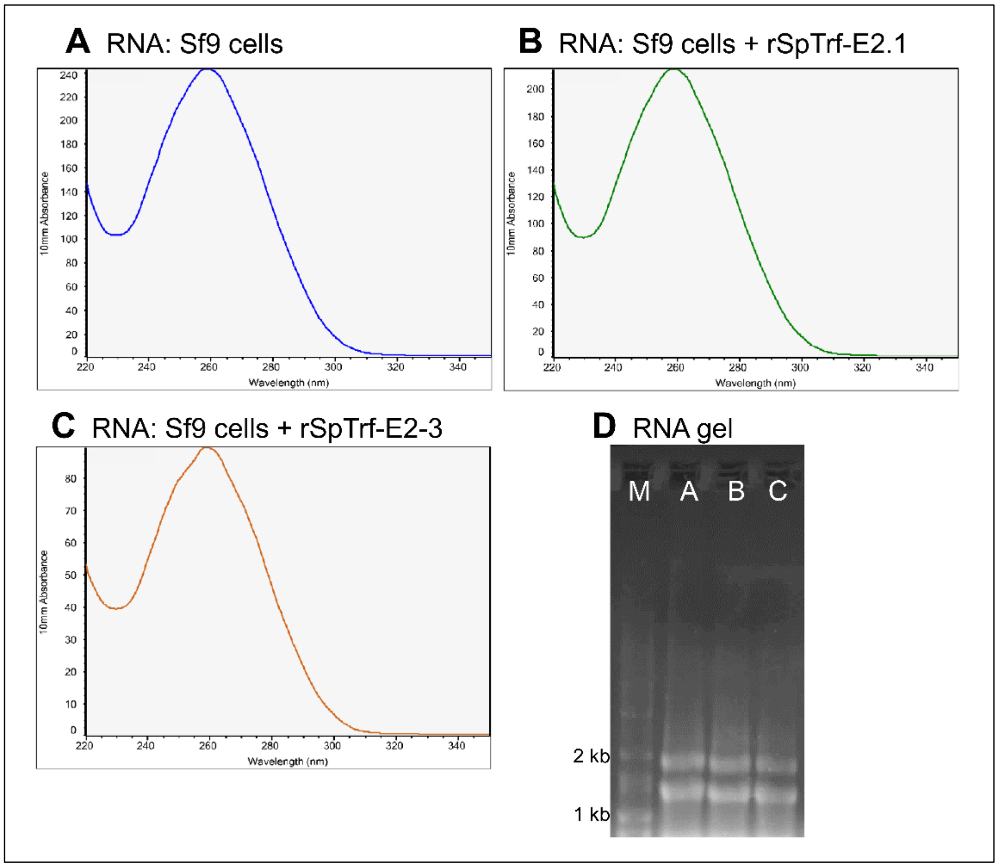

Fig. 1.4 | The *pMIB* expression constructs drive transcription and message accumulation in the Sf9 cells. Total RNA isolation for each cell culture was carried out according to the method for Trizol (Invitrogen). **(A-C)** Evaluation of the RNA concentration by spectrophotometry for OD^{260} (NanoDrop 2000c, ThermoFisher) indicates the presence of nucleic acids in all samples. **(D)** RNA from each sample was loaded onto a 0.8% agarose gel with TAE and ethidium bromide. Lane A, RNA (0.97 µg) from Sf9 cells; lane B , RNA (0.86 µg) from Sf9 cells transfected with *pMIB-E2.1* expression construct; lane C, RNA (0.36 µg) from Sf9 cells transfected with *pMIB-E2-3* expression construct. The bands at about 1.4 kb and 2.0 kb are tRNA and the smear is likely mRNA. Imaging under UV light was done in Kodak Molecular Imaging system (Kodak Gel Logic 1500). Standard DNA marker sizes are indicated (lane M).

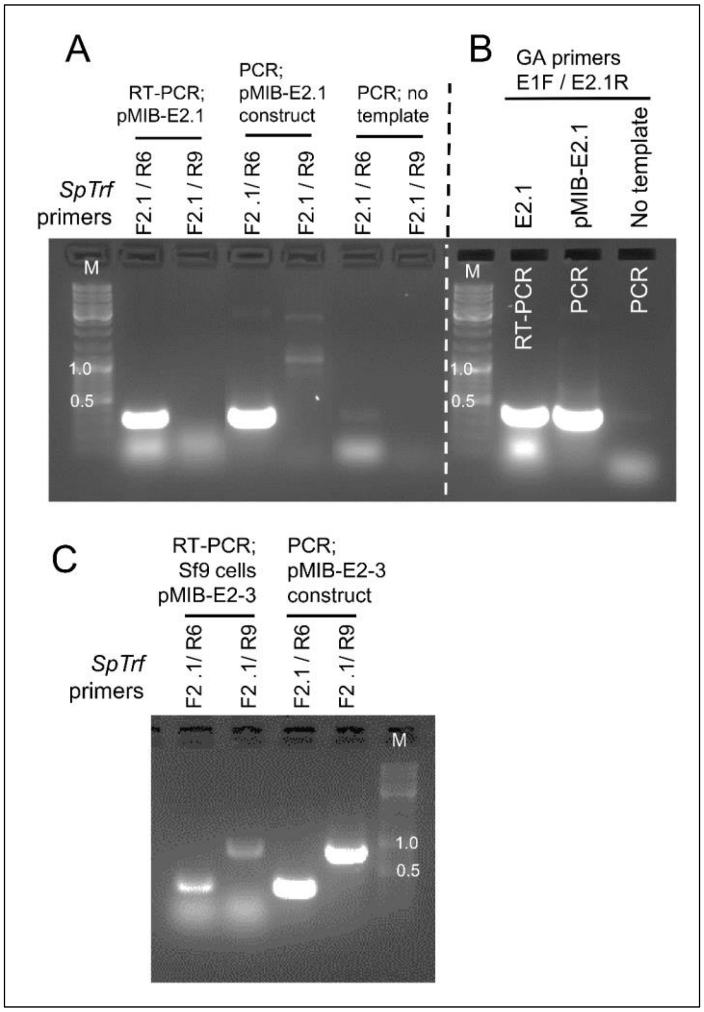

Fig. 1.5 | The *pMIB-E2.1* construct is expressed in Sf9 cells. **(A)** Reverse transcription of total RNA isolated from Sf9 cells transfected with *pMIB-E2.1* followed by PCR (RT-PCR) with the *F2.1*/*R6* primers (Table 1.1) shows that the cells contain mRNA from the expression vector. The amplicon from the Sf9 cells is the same size as the amplicon generated by PCR of the *pMIB-E2.1* construct. Amplification with the *F2.1*/*R9* primers fails because the *E2.1* sequence does not include the *R9* annealing site (see Fig. 1.3D). The gel image in **(A)** has been edited to remove duplicate lanes. **(B)** RT-PCR of total RNA isolated from Sf9 cells transfected with *pMIB-E2.1* with the primers used to amplify the cDNA insert for Gibson Assembly (GA) Cloning (*E1F*/*E2.1*(*pMIB*)*R*; Table 1.1), shows that the cells contain mRNA from the expression vector. The amplicon generated by PCR from the expression construct with the same GA primers is the same size. (Note that the *E1F* primer amplifies both *E1* and *E2* sequences.) **(C)** RT-PCR of total RNA isolated from Sf9 cells transfected with *pMIB-E2-3* with both *F2.1*/*R6* and *F2.1*/*R9* primers result in two sizes of amplicons. PCR with *pMIB-E2-3* results in the same two sizes of amplicons. This shows that the *F2.1*/*F9* primers function as expected. Relevant sizes in the GeneRuler DNA standards (ThermoFisher) are indicated. See Fig. 1.3D for the locations of the SpTrf primers.

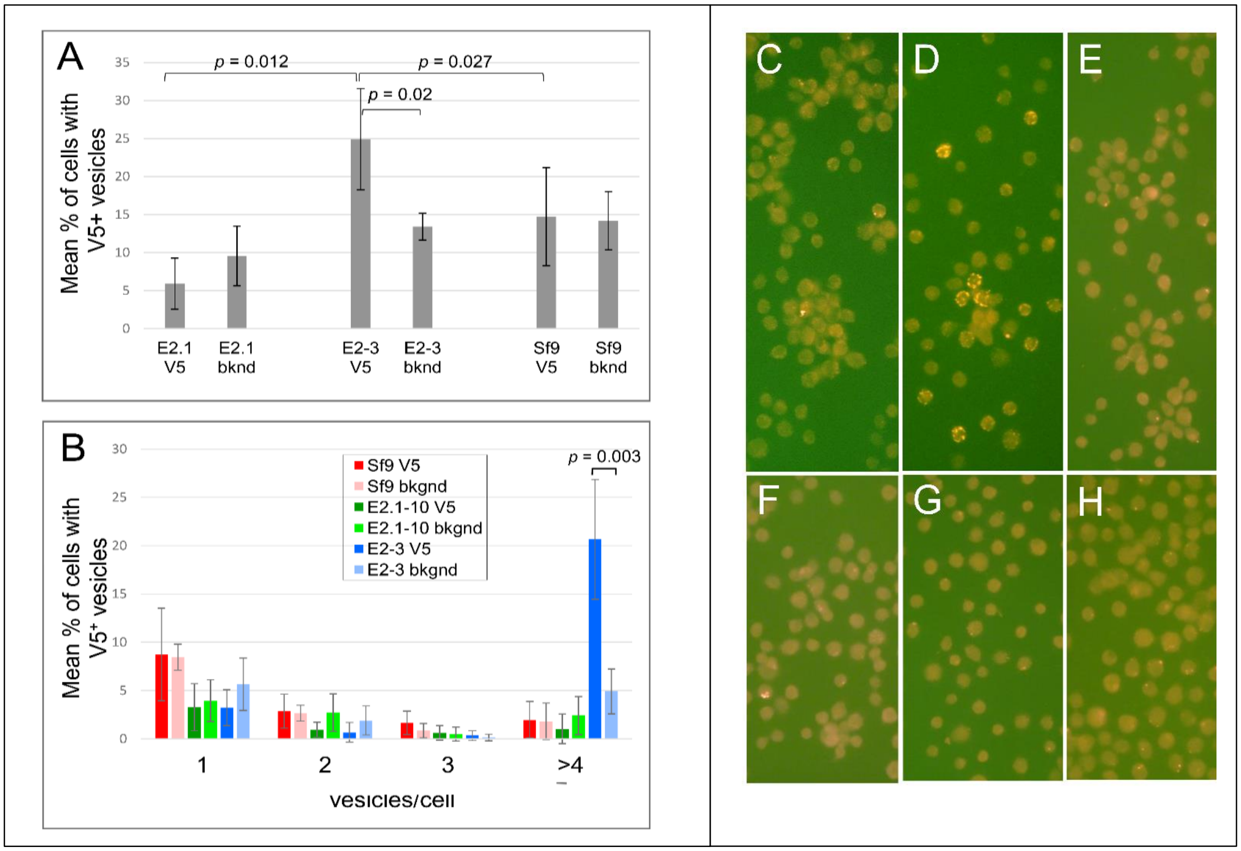

Fig. 1.6 | rSpTrf-E2.1 is not produced by Sf9 cells compared to rSpTrf-E2-3. Three cultures of Sf9 cells, un-transfected cells, cells transfected with the *pMIB-E2.1* construct, and cells transfected with the *pMIB-E2-3* construct, were incubated with Brefeldin A (5 µg/ml, BioLegend) for 18 hours at 27°C to block secretion. This treatment accumulated transport vesicles in the cells to augment differences among the three cultures. Cells were fixed and incubated with rabbit-anti-V5 or with normal rabbit serum followed by goat-anti-rabbit-Ig-549 (bkgnd, background). **(A)** The mean percentage of cells with one or more $V5^+$ vesicles is shown for each culture. The percentage of cells with rSpTrf-E2.1 in transport vesicles is not different from background, whereas the percentage of cells with rSpTrf-E2-3 in transport vesicles is significantly more than cells expressing rSpTrf-E2.1, or background levels in Sf9 cells. **(B)** Sf9 cells transfected with *pMIB-E2.1* do not produce rSpTrf-E2.1 proteins. The number of $V5^+$ vesicles per cell for Sf9 cells transfected with *pMIB-E2.1* is not different from cells that were not transfected with an expression vector. In contrast, cells transfected with *pMIB-E2-3* have significantly more $V5^+$ vesicles compared to background. Significance for all comparisons was established by the two tailed *t*-test ($p < 0.05$). **(C-H)** Results in **(B)** were obtained from fluorescence microscopy images of cells with $V5^+$ vesicles **(C-E)** compared to background **(F-H)**. **(C, F)** Cells transfected with rSpTrf-E2.1, **(D, G)** cells transfected with rSpTrf-E2-3, **(E, H)** un-transfected Sf9 cells.

Table for Supplementary Text File 1

Table 1.1 | Primers used in PCR

Primer	Sequence	Tm, ºC
E1(pMIB)F[1]	gtatacatttcttacatctatgccCAAAGAGATTTCAATGAACGACG	65.4
E2.1(pMIB)R[2]	gggttagggagaggcttaccATGACCATCAAACTGACCGCG	71.5
E2(pMIB)R[3]	gggttagggagaggcttaccAGGTGGTGAAGATGTCGTTAC	70.5
pMIBF[4]	GCGCGCCTATAAATACAGC	56.0
pMIBR[4]	GACAATACAAACTAAGATTTAGTCAG	54.4
CyF2[5]	GTGACGACGATGTTGCCGC	64.5
CyR2[5]	TTGGGGTTGAGGGGAGCC	64.5
SpTrf-F2.1[6]	AGMGATTWCAATGAACKRCGAGGA	62.0
SpTrf-R6[6]	CGAGCATCAGTTTCTTTCKTCTC	61.7
SpTrf-R9[6]	CTTHARGTGGTGGAARATGTCG	59.3

[1]This forward primer anneals to the 5' end of 2-2439 cDNA [3], and was designed to generate a 5' ligation region for cloning into the *pMIB* vector by Gibson Assembly (New England BioLabs). It is used to amplify both *E1* and *E2* sequences. Lower case nucleotides indicate the sequence match to *pMIB*. Upper case nucleotides indicate the sequence match to the *SpTrf* insert sequence.

[2]This reverse primer anneals to the middle of 2-2439 cDNA to amplify the truncated version of *E2*, termed *E2.1*. The primer was designed to produce a 3' ligation site for cloning into the *pMIB* vector by Gibson Assembly. Lower case nucleotides indicate the sequence match to *pMIB*. Upper case nucleotides indicate the sequence match to the SpTrf insert sequence.

[3]This primer anneals to the 3' end of the coding region of 2-2436 cDNA amplify the full length *E2* insert. It was designed for cloning into the *pMIB* vector by Gibson Assembly. Lower case nucleotides indicate the sequence match to *pMIB*. Upper case nucleotides indicate the sequence match to the SpTrf insert sequence.

[4]This primer pair surrounds the insert and was used to amplify all *SpTrf* inserts.

[5]*Cy*, cytoplasmic actin.

[6]See Fig. 1.3D for the locations of the *SpTrf* primers.

Supplementary Text File S2

The initial approach to evaluate phagocytosis of rSpTrf::beads by sea urchin phagocytes used magnetic attraction of cells associated with beads

The functions of the natSpTrf proteins were originally speculated to act as opsonins that bind to foreign cells and PAMPs to augment phagocytosis. The first functional characterization of a recombinant SpTrf protein had an E1 element pattern, rSpTrf-E1, and it bound to *Vibrio diazotrophicus* [12], but did not augment its phagocytosis by sea urchin coelomocytes [11]. It was proposed that this might be a characteristic of natSpTrf proteins with an E1 element pattern, and that other natSpTrf isoforms would function differently, resulting in an effective immune protection system. To test this hypothesis, cells that phagocytosed magnetic beads that were cross-linked to different molecules or left non cross-linked, were evaluated after attraction to a magnet, as has been reported for isolating phagosomes from phagocytes [13]. CF was collected from sea urchins in calcium- and magnesium-free sea water with EDTA and HEPES buffer (CMFSW-EH; 460 mM NaCl, 10.7 mM KCl, 7.04 mM Na_2SO_4, 2.38 mM $NaHCO_3$, 70 mM EDTA, 20 mM HEPES pH 7.4; [14]) and the cells were pelleted at 500 x *g* for 5 min and resuspended in coelomocyte culture medium (CCM; 0.5 M NaCl, 5 mM $MgCl_2$, 1 mM EGTA, 20 mM HEPES, pH 7.4; [2, 15]). rSpTrf-E2-3, rSpTrf-E2-4, and BSA were cross-linked to 1 µm magnetic COOH-beads (Bangs Laboratories) by the 2-step EDAC cross-linking method (Polysciences). BSA cross-linked to COOH-beads (BSA::beads) served as the negative control. Streptavidin (SA)-beads (Bangs Laboratory) were incubated with LPS-biotin (LPS::beads) from *E. coli* (0111:B4 strain; Invivogen) according to the manufacturer (Bangs Laboratory) and served as the positive control. Control SA-beads were processed similarly, but LPS-biotin was omitted. Beads were incubated in solution with coelomocytes (50:1) in 500 µl CCM at 14°C for 20 min. Cells containing beads were collected by magnetic attraction for 15 min on ice. The supernatant was moved to a new tube, which contained cells that were not attracted to the magnet, and cells in the capture fraction were washed once with CCM, and resuspended into 50 µl CCM. The average number of beads per cell was evaluated by microscopy and significant differences in the average number of beads per cell among the different cross-linked beads were identified with a Tukey ANOVA test. Results showed that the average number of beads per cell was significantly higher for LPS::beads, and beads cross-linked to rSpTrf-E2-3, and -E2-4, compared to COOH-beads (Fig. 2.1). However there was no significant difference between the LPS::beads, rSpTrf-E2-3::beads, and the rSpTrf-E2-4::beads compared to the BSA::beads. This suggested a problem with the assay because the average number of LPS::beads (positive control) per cell was not different from the BSA::beads (negative control).

To determine whether this unexpected result was an outcome of the relatively short incubation time for cells to interact with beads (20 min), the experiment was repeated using several time points (0, 10, 20, 60, 90 min), and followed the same magnetic capture method described above. This was undertaken to determine whether longer incubation periods would reveal differences for cells with phagocytosed beads. Cell were counted for each fraction using microscopy, and the average percentage of cells collected by the magnet was calculated based on the total number of cells in both the magnet capture fraction and the supernatant. Results showed that for incubations with each bead treatment, the average percentage of cells attracted to the magnet remained unchanged and did not increase at longer time periods (Fig. 2.2). At the 90 min time point, the percentage of cells incubated with BSA::beads was lower than at 0 min. Furthermore, there were cells present in the magnet capture fraction at 0 min for all bead

treatments, which was unexpected and unlikely that the cells had time to phagocytose beads. When the cell types were evaluated by microscopy, there were phagocytes with beads in the magnet capture fraction, but there were also phagocytes without beads. Furthermore, this fraction contained all types of coelomocytes without beads including red and colorless spherule cells and vibratile cells that are not known to be phagocytic (reviewed in [16, 17]). These results suggested that all types of coelomocytes bound to the walls of the plastic tubes and that the assay did not evaluate phagocytosis. The results also suggested that cells that had phagocytosed beads and were attracted to the magnet were subsequently mixed with cells that had bound to the tube wall during incubations and included cells without beads. Consequently, the outcome identified a large background effect that confounded attempts to characterize phagocytosis of beads cross-linked to different molecules.

To confirm whether any cells captured by the magnet had phagocytosed beads, cells were pre-treated with cytochalasin D (cytoD, 32 µM; SigmaAldrich) for 30 minutes. This treatment inhibited actin polymerization and consequently blocked phagocytosis. Following the above protocol cytoD treated and un-treated cells were incubated with COOH-beads for 20 min and the cells in both the magnet and supernatant fractions were counted using microscopy. Cells were found in the magnet capture even when treated with cytoD (Fig. 2.3). Furthermore, cytoD treated cells did not show significant differences in the average percentage of cells in the magnet capture fraction compared to cells that were not treated with cytoD. These results indicated that the magnet based capture and analysis of cells that had bound and/or phagocytosed magnetic beads was not feasible for sea urchin coelomocytes because all cell types bound to the plastic tube walls. Consequently, an alternative method was developed to incubate the cells with beads followed by evaluation by microscopy. Because the phagocytes bound tightly to glass, whereas other coelomocyte types did not and were removed by washes, the evaluation of phagocyte detection and phagocytosis of cross-linked beads was employed (see the main paper).

Figures for Supplementary Text File 2

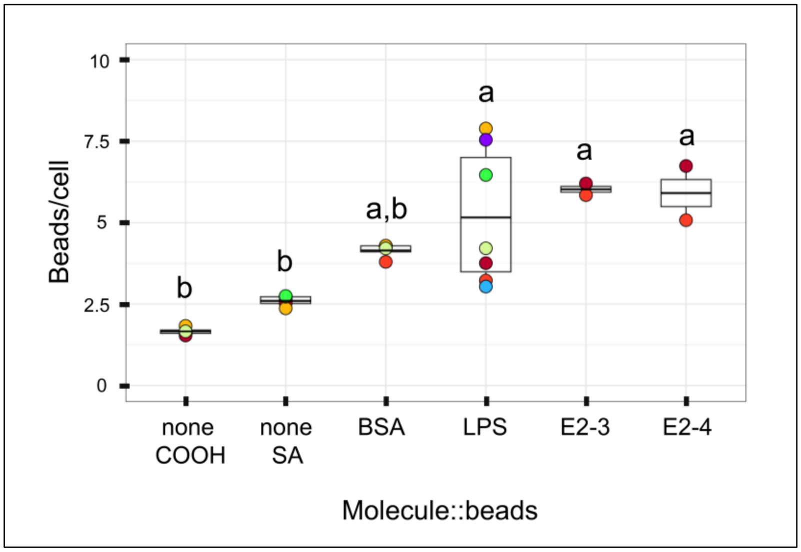

Fig. 2.1 | Phagocytes do not differentiate among beads cross-linked with rSpTrf proteins, LPS, or BSA. Cells were incubated with beads for 20 min and the number of beads per cell was evaluated for cells captured by the magnet. COOH (beads not cross-linked to a molecule), SA (beads not cross-linked to a molecule). Beads cross-linked with BSA, LPS, or rSpTrf proteins are indicated. The box plots show the average and interquartile range of each bead treatment associated with phagocytes and results for cells from different sea urchins are identified by colored dots. Significant differences were determine by a Tukey ANOVA test (see Methods section 2.9 in the main paper). The letters above the boxplots indicate significant differences and similarities.

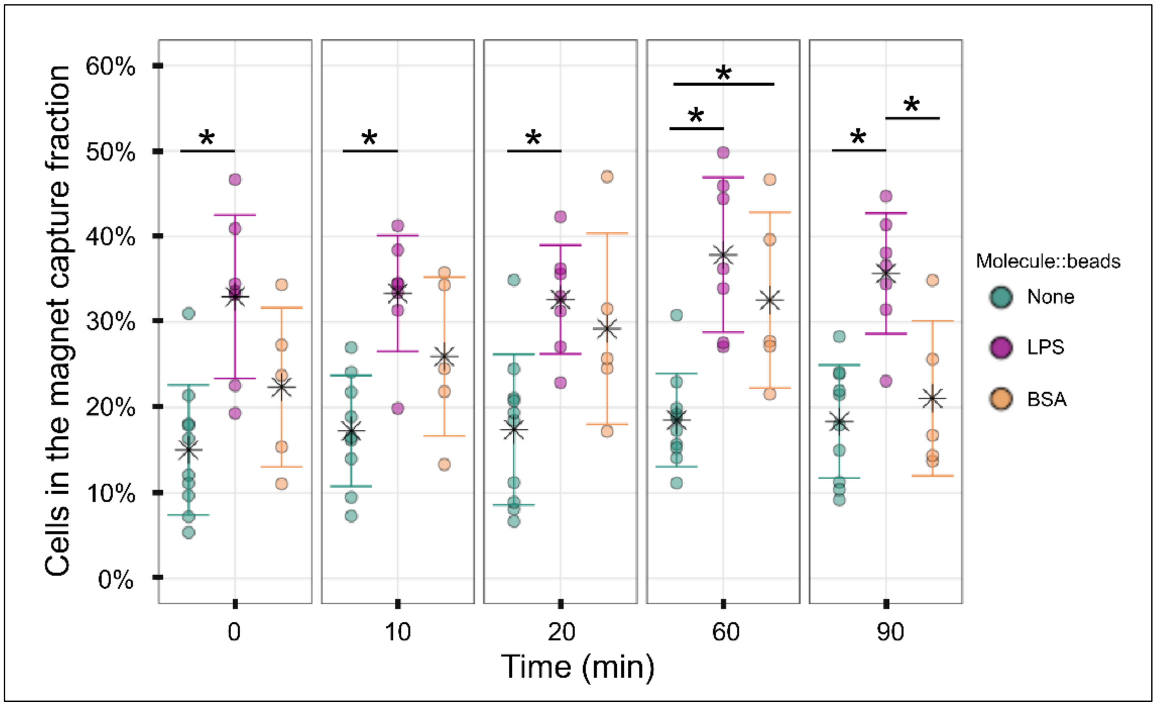

Fig. 2.2 | Cells in the magnet capture fraction are present at all time points. The average number of cells that were associated with beads cross-linked to LPS or BSA or non-cross-linked COOH-beads was evaluated by microscopy. Black stars indicate the average number of cells in the magnet capture fraction, and error bars show the standard deviation for each bead type tested. Significant differences were determined using a Tukey ANOVA test for each time point. Results are approximately the same for the three bead types across all time points, including results at 0 min. *, $p < 0.05$.

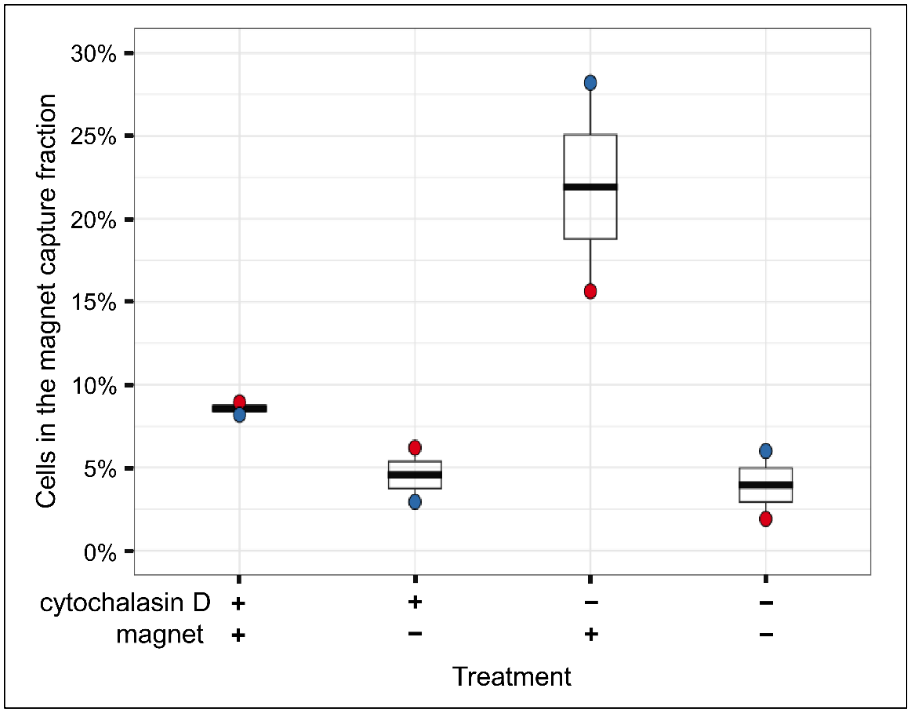

Fig. 2.3 | Coelomocytes are present in the magnet capture fraction irrespective of treatment with cytochalasin D. Cells incubated with cytochalasin D to block phagocytosis followed by incubation with COOH-beads are present in both the magnet capture fraction and the supernatant containing cells that are not attracted by the magnet. Omitting cytochalasin D did not show significant changes to the percentage of cells in the magnet capture fraction. The box plots show the average and interquartile range of each treatment associated with phagocytes, and none of the treatments are significantly different based on a Tukey ANOVA test (see Methods section 2.9 in the main paper).

References

1. Lun CM, Samuel RL, Gillmor SD, Boyd A, Smith LC. The recombinant sea urchin immune effector protein, rSpTransformer-E1, binds to phosphatidic acid and deforms membranes. Frontiers in Immunology. 2017;8:481. doi: 10.3389/fimmu.2017.00481
2. Brockton V, Henson JH, Raftos DA, Majeske AJ, Kim YO, Smith LC. Localization and diversity of 185/333 proteins from the purple sea urchin - unexpected protein-size range and protein expression in a new coelomocyte type. Journal of Cell Science. 2008;121(3):339-48. doi: 10.1242/jcs.012096
3. Terwilliger DP, Buckley KM, Brockton V, Ritter NJ, Smith LC. Distinctive expression patterns of *185/333* genes in the purple sea urchin, *Strongylocentrotus purpuratus*: an unexpectedly diverse family of transcripts in response to LPS, beta-1,3-glucan, and dsRNA. BMC Molecular Biology. 2007;8:16. doi: 10.1186/1471-2199-8-16
4. Buckley KM, Terwilliger DP, Smith LC. Sequence variations in *185/333* messages from the purple sea urchin suggest posttranscriptional modifications to increase immune diversity. Journal of Immunology. 2008;181:8585-94. doi: 10.4049/jimmunol.181.12.8585barella
5. Dheilly NM, Nair SV, Smith LC, Raftos DA. Highly variable immune-response proteins (185/333) from the sea urchin *Strongylocentrotus purpuratus*: proteomic analysis identifies diversity within and between individuals. Journal of Immunology. 2009;182:2203-12. doi: 10.4049/jimmunol.07012766
6. Sherman LS, Schrankel CS, Brown KJ, Smith LC. Extraordinary diversity of immune response proteins among sea urchins: nickel-isolated Sp185/333 proteins show broad variations in size and charge. PLoS ONE. 2015;10(19):e0138892. doi: 10.1371/journal.pone.0138892
7. Smith LC, Lun CM. The *SpTransformer* gene family (formerly *Sp185/333*) in the purple sea urchin and the functional diversity of the anti-pathogen rSpTransformer-E1 protein. Frontiers in Immunology. 2017;8:725. doi: 10.3389/fimmu.2017.00725
8. Hossainey MRH, Yaparla A, Uzzaman Z, Moore T, Grayfer L. A comparison of amphibian (*Xenopus laevis*) tadpole and adult frog macrophages. Developmenal and Comparative Immunology. 2023;141:104647. doi: 10.1016/j.dci.2023.104647
9. Hossainey MRH, Yaparla A, Hauser KA, Moore TE, Grayfer L. The roles of amphibian (*Xenopus laevis*) macrophages during chronic frog virus 3 infections. Viruses. 2021;13(11):2299. doi: 10.3390/v13112299
10. Lovelace P, Maecker HT. Multiparameter intracellular cytokine staining. In: Hawley T, Hawley, R., editor. Flow Cytometry Protocols Methods in Molecular Biology. Methods in Molecular Biology. 3rd ed. Totowa, NJ, USA: Humana Press; 2011. p. 165-78. doi: 10.1007/978-1-61737-950-5_8
11. Chou H-Y, Lun CM, Smith LC. The SpTransformer proteins from the purple sea urchin opsonize bacteria, augment phagocytosis, and retard bacterial growth. PLoS One. 2018;13(5):e0196890. doi: 10.1371/journal.pone.0196890
12. Lun CM, Schrankel CS, Chou H-Y, Sacchi S, Smith LC. A recombinant Sp185/333 protein from the purple sea urchin has multitasking binding activities towards certain microbes and PAMPs. Immunobiology. 2016;221:889-903. doi: 10.1016/j.imbio.2016.03.006
13. Pryor PR, P. RA. Isolating phagosomes from tissue culture cells. Cold Spring Harbor Protocols. 2014;12:1320-3. doi: 10.1101/pdb.prot074468

14. Smith LC, Hawley TA, Henson JH, Majeske AJ, Oren M, Rosental B. Methods for collection, handling, and analysis of sea urchin coelomocytes. In: Foltz K, Hamdoun A, editors. Methods in Cell Biology. 150, part A: Elsevier; 2019. doi: 10.1016/bs.mcb.2018.11.009
15. Henson JH, Svitkina TM, Burns AR, Hughes HE, MacPartland KJ, Nazarian R, et al. Two components of actin-based retrograde flow in sea urchin coelomocytes. Molecular Biology of the Cell. 1999;10(12):4075-90. doi: 10.1091/mbc.10.12.4075
16. Smith LC, Ghosh J, Buckley KM, Clow LA, Dheilly NM, Haug T, et al. Echinoderm immunity. In: Soderhall K, editor. Invertebrate Immunity. Advances in Experimental Medicine and Biology. 708. Austin Texas: Landes Bioscience and Spring Science+Business Media; 2010. p. 260-301. doi: 10.1007/978-1-4419-8059-5_14
17. Smith LC, Arizza V, Barela Hudgell MA, Barone G, Bodnar AG, Buckley KM, et al. Echinodermata: The complex immune system in echinoderms. In: Cooper E, editor. Advances in Comparative Immunology: Springer Publisher; 2018. p. 409-501.
